# Deducing the internal interfaces of twisted multilayer graphene via moiré-regulated surface conductivity

**DOI:** 10.1093/nsr/nwad175

**Published:** 2023-06-19

**Authors:** Huan Wang, Sen Wang, Shuai Zhang, Mengzhen Zhu, Wengen Ouyang, Qunyang Li

**Affiliations:** Applied Mechanics Laboratory, Department of Engineering Mechanics, Tsinghua University, Beijing 100084, China; Department of Engineering Mechanics, School of Civil Engineering, Wuhan University, Wuhan 430072, China; Applied Mechanics Laboratory, Department of Engineering Mechanics, Tsinghua University, Beijing 100084, China; Applied Mechanics Laboratory, Department of Engineering Mechanics, Tsinghua University, Beijing 100084, China; Department of Engineering Mechanics, School of Civil Engineering, Wuhan University, Wuhan 430072, China; State Key Laboratory of Water Resources and Hydropower Engineering Science, Wuhan University, Wuhan 430072, China; Applied Mechanics Laboratory, Department of Engineering Mechanics, Tsinghua University, Beijing 100084, China; State Key Laboratory of Tribology in Advanced Equipment, Tsinghua University, Beijing 100084, China

**Keywords:** twisted graphene, electrical conductivity, embedded interface, atomic reconstruction, moiré pattern

## Abstract

The stacking state of atomic layers critically determines the physical properties of twisted van der Waals materials. Unfortunately, precise characterization of the stacked interfaces remains a great challenge as they are buried internally. With conductive atomic force microscopy, we show that the moiré superlattice structure formed at the embedded interfaces of small-angle twisted multilayer graphene (tMLG) can noticeably regulate surface conductivity even when the twisted interfaces are 10 atomic layers beneath the surface. Assisted by molecular dynamics (MD) simulations, a theoretical model is proposed to correlate surface conductivity with the sequential stacking state of the graphene layers of tMLG. The theoretical model is then employed to extract the complex structure of a tMLG sample with crystalline defects. Probing and visualizing the internal stacking structures of twisted layered materials is essential for understanding their unique physical properties, and our work offers a powerful tool for this via simple surface conductivity mapping.

## Teaser

Embedded structures in layered materials can be seen through by taking a map of surface conductivity in a way similar to X-ray imaging.

## INTRODUCTION

Vertically stacking two-dimensional (2D) materials to introduce strong interlayer coupling is a powerful approach for regulating their physical properties [1–4]. In particular, one can tune the interlayer coupling and influence the electronic properties of the stacked structures by twisting the interface [5–9], which has spawned a new field: twistronics [6,10]. For example, when two graphene monolayers are vertically stacked with a small twist angle, the two monolayers will undergo spontaneous atomic reconstruction due to the competition between the interlayer van der Waals (vdW) interaction and the intralayer elasticity [11–16], leading to a rearrangement of atoms to form domains with locally commensurate stacking and strained soliton boundaries. This special moiré superlattice structure caused by atomic reconstruction can significantly modulate the vibration modes [17], electronic structures [18,19] and electron–phonon coupling behavior [20] of the system and lead to electronic reconstruction [11] with many unexpected phenomena [15,21–28]. In addition to the well-known twisted bilayer graphene (tBLG) structure, richer physical behaviors with more peculiar effects have been discovered for twisted multilayer systems. Recent studies have shown that some new correlated features have emerged in twisted multilayer structures, such as the strong tunability of the band structure via a vertical electric field [29,30], spin-polarized ground states [31], perfect minivalley polarization [32] and controllable thermal conductivity [33]. Despite the interesting properties of these twisted multilayer systems, the detailed structure of the embedded twisted interfaces and their impact on neighboring atomic layers as well as all the stacked units remain less understood.

Previously, it has been shown that the surface electrical conductivity measured by conductive atomic force microscopy (c-AFM) was very sensitive to the stacking states of graphene when the topmost layer was twisted and became reconstructed [34–36]. In this study, we explored the surface conductivity of twisted multilayer graphene (tMLG) structures and found that the twisted interfaces could notably affect surface conductivity even when they were embedded 10 atomic layers beneath the surface. Assisted by molecular dynamics (MD) simulations, we propose a theoretical model, namely the series spreading resistance (SSR) model, to quantitatively correlate the surface conductivity of tMLG systems with their internal atomic structures. The findings provide a route to visualizing and quantifying the atomic stacking states of embedded interfaces from a simple surface conductivity measurement, which was validated even for complex tMLG structures with defects such as dislocations.

## RESULTS

**Figure 1.**
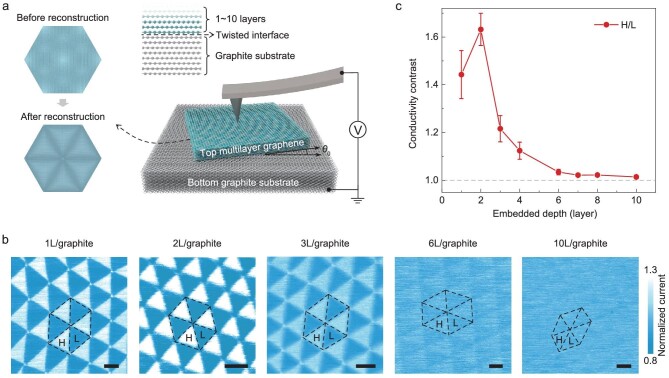
Experimental characterization of surface conductivities on tMLG samples with twisted interface embedded at different depths. (a) A schematic diagram showing c-AFM measurements on the tMLG surface (right panel) where a constant voltage bias is applied between the conductive tip and the bottom graphite. The figure also shows schematics of the atomic structures of the two atomic layers at the twisted interface before (upper-left panel) and after (lower-left panel) reconstruction. (b) Typical current maps obtained from the twisted 1 L/graphite (with a twist angle of 0.16° ± 0.02°), 2 L/graphite (0.25° ± 0.01°), 3 L/graphite (0.20° ± 0.02°), 6 L/graphite (0.13° ± 0.02°) and 10 L/graphite (0.21° ± 0.04°). The reported current maps are normalized by the averaged current values of the corresponding images. The different stacking regions are highlighted with black dotted triangles, where L and H represent the low and high conductivity domains. Scale bars, 50 nm. (c) The current ratio between H domain and L domain as a function of the embedded depth of the twisted interface for different tMLG samples. The embedded depth can also be expressed as the number of top graphene layers above the twisted interface. The error bar represents the standard deviation of different samples or different current images.

A schematic diagram of the electrical conductivity measurements of tMLG devices is shown in Fig. 1a. For the small-angle twisted interface, previous studies of tBLG [11–14] have shown that the competition between the vdW interaction energy and elastic strain energy leads to atomic reconstruction, resulting in rotation of local domains thereby forming a periodic strain soliton network, as illustrated in the left inset of Fig. 1a. However, for the embedded twisted interface in tMLG, the two graphene layers at the twisted interface are constrained by surrounding graphene layers. It is not clear whether the reconstruction will still occur or not. Furthermore, it has been shown that, when the topmost graphene is reconstructed, the surface conductivity will exhibit a different contrast due to the alternating ABA- and ABC-stacked structures [35]. If reconstruction can also occur for the embedded twisted interface, how it affects the surface conductivity of tMLG is scientifically intriguing and remains unexplored. To answer these questions, we performed c-AFM experiments to characterize the surface conductivity of tMLG by measuring the current flowing from the bottom graphite substrate through the twisted interface and the top multilayer graphene and finally to the probe (see details in Methods and Fig. S1).

Figure 1b shows five typical current maps measured on tMLG samples with the number of top graphene layers varying between 1, 2, 3, 6 and 10 (results for other numbers of layers and raw current data can be found in Figs S5–S7). It can be seen that the current signals on all tMLG surfaces show a triangular pattern, although the contrast is relatively weak when the twisted interface is embedded 10 layers beneath the surface (sample 10 L/graphite). This alternating bright and dark triangular arrangement indicates the occurrence of periodically alternating stacking states originating from local atomic reconstruction. Thus, our experimental results directly show that reconstruction can still occur for the twisted interface embedded in the interior of a material. In addition, as the embedded depth of the twisted interface increases, the conductivity contrast between different domains becomes weaker from the surface conductivity measurements (with the exception of the 2 L/graphite sample). Figure 1c summarizes the average current ratio of the high-conductivity triangular domain to the low-conductivity domain as a function of the depth of the embedded interface. It can be seen that the ratio increases from 1.44 at 1 L/graphite to 1.63 at 2 L/graphite, reaching the maximum at 2 L/graphite, and then decreases almost exponentially with increasing embedded depth. It should be noted that the conductivity contrast between different domains barely changes as the twist angle varies from 0.11° to 0.29° (Fig. S8). Since c-AFM mainly measures the spreading resistance, this measurement technique is quite sensitive to the mean conductivity within a volume near the surface. Therefore, when the twisted interface is embedded at a higher depth, we expect that the changes in stacking order caused by reconstruction may have a weaker effect on surface conductivity, resulting in a lower contrast between different domains. Our measurements suggest that the impact of the twisted interface on surface conductivity can propagate roughly as far as 10 graphene layers. It is worth mentioning that when the twisted interface is embedded under two-layer graphene (2 L/graphite), there is an unusual increase in surface conductivity contrast compared to the case of a 1 L/graphite structure. This non-monotonic trend should be related to the specific stacking structures of the tMLG.

**Figure 2.**
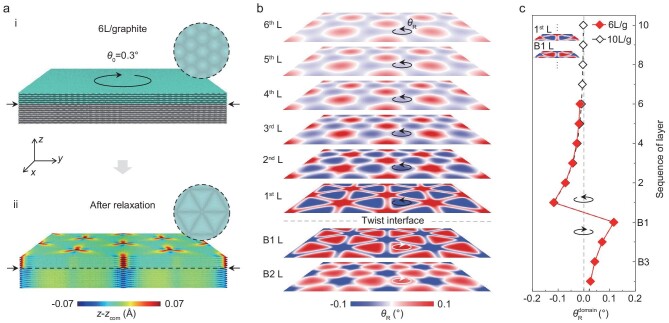
MD simulation results. (a) An example MD model of 6 L/graphite (i) and the height undulation of the system after relaxation (ii). Insets show the snapshots of the two graphene layers adjacent to the twisted interface before (i) and after (ii) relaxation. (b) Maps of the atomic rotation angle ${\theta }_{\rm{R}}$ in individual graphene layers. ${\theta }_{\rm{R}}$ is obtained by calculating the relative rotation angle before and after relaxation. Positive ${\theta }_{\rm{R}}$ means rotation in the direction of the twist angle and negative ${\theta }_{\rm{R}}$ means the counter-twist direction. (c) Variations of $\theta _{\rm{R}}^{{\rm{domain}}}$ in different layers of the 6 L/graphite and 10 L/graphite systems. The reported $\theta _{\rm{R}}^{{\rm{domain}}}$ are the averaged values of ${\theta }_{\rm{R}}$ for all atoms around the center area of the AB/BA domains (within a radius of 2 nm), as marked in the inset of panel (c).

Since directly tracking the detailed reconstruction state of different layers embedded inside the samples by experiment is extremely challenging, to better understand the atomic structures of tMLG, we conducted MD simulations of tMLG devices containing top AB-stacked graphene slabs with thicknesses varying from 1 to 10 layers deposited on an AB-stacked thick graphite substrate (see details in the section of MD simulation of the atomic reconstruction process and Figs S9–S17 of the supplementary data). A typical MD model consisting of a 6 L/graphite composite structure is shown in Fig. 2. In this model, a 10-layer graphene slab is used to simulate the bottom graphite (the lower layers in Fig. 2a(ⅰ)), and a 6-layer graphene slab (the upper layers in Fig. 2a(i)) is twisted by an initial angle ${\theta }_0$ of 0.3° and placed on the bottom graphite. Figure 2a(ii) shows the relaxed atomic configuration of the 6 L/graphite structure. As shown by the inset of Fig. 2a(ii), clear triangular domains can be observed at the twisted interface, indicating that significant atomic reconstruction indeed occurs at the interface. Meanwhile, the reconstruction also causes out-of-plane displacement of the graphene layers, which can propagate to the outermost layer with gradually decaying amplitudes (Fig. S17); similar behavior has been observed for graphene/*h*-BN heterostructures [16]. Further calculations confirm that atomic reconstruction actually occurs at the twisted interface for all tMLG samples (1∼10 L/graphite) (see Figs S12–S17 for more details).

To quantitatively study interfacial reconstruction and its impact on the internal structure of tMLG, we compared the distributions of atomic in-plane rotation angles ${\theta }_{_{\rm{R}}}$ (the definition of ${\theta }_{_{\rm{R}}}$ is shown in Fig. S11) within each layer of the 6 L/graphite structure. As shown in Fig. 2b, ${\theta }_{_{\rm{R}}}$ of the two graphene layers adjacent to the twisted interface, i.e. the 1st L and B1 L, is the largest; and the twist directions of the 1st L and B1 L are opposite, resulting in nearly AB- or BA-stacking in the reconstructed triangular domains. This in-plane deformation behavior can be understood as follows. Because the commensurate stacking (AB- or BA-stacking) is energetically more favorable than the incommensurate states, the graphene lattice at the twisted interface tends to undergo local in-plane deformation (i.e. reconstruction) to achieve better commensuration. Therefore, the AB- or BA-stacked domains are expected to grow due to minimization of the interfacial stacking energy. However, as the interface is twisted, the local in-plane deformation within individual domains is different and the expansion of the domains results in significant strain in the domain wall regions. The excess strain energy of the domain walls counteracts the interfacial energy to alleviate the in-plane lattice deformation to reduce the degree of atomic reconstruction. The interplay of these two energies determines the final reconstruction state of the interface [11–14].

The in-plane rotation of the 1st L and B1 L at the twisted interface is also found to promote the in-plane rotational deformation of the adjacent graphene layers (see Supplementary Movie). For example, rotation of the 1st L will cause rotational deformation of the 2nd L, and similarly, rotation of the 2nd L will induce rotational deformation of the 3rd L and so on. This suggests that any of the two adjacent layers within the tMLG structure still tend to maintain the AB-stacking state to minimize the interfacial energy when the internal twisted interface is reconstructed. Despite this tendency, our MD simulation results indicate that atomic rotation angle ${\theta }_{_{\rm{R}}}$ of the graphene layers gradually decays when they are away from the interface. For example, as shown in Fig. 2c, the ${\theta }_{_{\rm{R}}}^{{\rm{domain}}}$ (the averaged ${\theta }_{_{\rm{R}}}$ values around the center area of the AB/BA domains) of the graphene layer at six layers from the interface (6th L) has dropped to 7% compared with the 1st L. This decaying trend in the in-plane rotation angle suggests that the multilayer graphene structure imposes a certain constraint on the reconstruction behavior of the twisted interface. This is confirmed by the fact that the ${\theta }_{_{\rm{R}}}^{{\rm{domain}}}$ of the 1st L in 10 L/graphite is slightly smaller than that of the 1st L in 1 L/graphite (Fig. S17). However, we found that the effect of this constraint was rather limited for tMLG with small twist angles.

**Figure 3.**
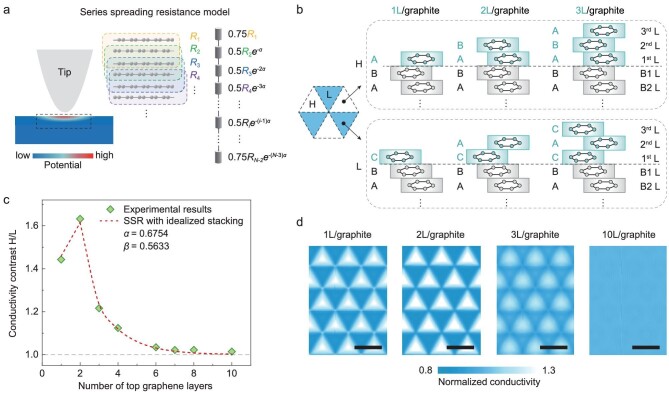
A theoretical model for estimating the surface conductivity of tMLG. (a) A schematic of the SSR model for calculating the surface conductivity of a multilayer structure. Schematic distribution of the electrical potential during a typical c-AFM experiment (left panel); the definition of *R_i_* (middle panel); SSR model with a decaying factor *e*^−^*^α^* (right panel). (b) Schematics showing the idealized stacking structures for H domains and L domains in twisted 1 L/graphite, 2 L/graphite and 3 L/graphite structures; these stacking states are qualitatively confirmed by MD simulations. (c) Comparison between the theoretical prediction with idealized stacking configurations and the experimental results. (d) Calculated 2D conductivity maps of the twisted 1 L/graphite, 2 L/graphite, 3 L/graphite and 10 L/graphite structures with the same twist angles of 0.3° based on the local registry index. Scale bars, 50 nm.

Based on the atomic structures revealed in MD simulations, we proposed a theoretical model, the SSR model, to quantify the influence of the stacking state of tMLG on its surface conductivity. It is known that the measured current signal in c-AFM is determined by the local spreading resistance of the sample, which is closely associated with the resistivity in a nanoscale volume within the topmost graphene layer in tMLG, as schematically shown in Fig. 3a [37,38]. In our SSR model, the overall effective spreading resistance is approximated as a series of equivalent resistances *R_i_* along the sample thickness direction. Because of the nature of the spreading resistance, materials that are far away from the probe contribute less to the measured resistance. Such a decaying feature along the thickness direction is more prominent for tMLG due to the extremely high in-plane electrical conductivity of the graphene layer [39]. To capture this effect, an exponential decaying factor *e*^−^*^α^* was introduced to consider the contributions of layers at different depths to the overall resistance. Previous density functional theory (DFT) calculations have shown that the surface conductivity of the twisted monolayer/multilayer graphene is largely determined by the top three layers, and the effect of adding more AB-stacked bottom layers is limited [35]. Therefore, *R_i_* (*i* = 1, 2, 3…) in our model represents the equivalent resistance of each unit cell of three neighboring graphene layers, as shown in Fig. 3a. By adding *R_i_* in series, we could estimate the effective spreading resistance for a tMLG structure with *N* graphene layers to be


(1) \begin{eqnarray*} R &=& \,0.75{R}_1 + \sum_{i = 2}^{N - 3} {0.5{R}_i{e}^{ - \alpha (i - 1)}}\\ && +\, 0.75{R}_{N - 2}{e}^{ - \alpha (N - 3)}, \end{eqnarray*}

where the numbers in front of *R_i_*, i.e. 0.75 and 0.5, are factors to account for the repeated calculation (see more details in Fig. S18).

Although the MD calculations suggest that the in-plane rotation angle within the domains of individual graphene layers gradually decays (Fig. 2c), the deviation angle of any two adjacent graphene layers relative to the ideal AB- or BA-stacking is still <0.07°. To simplify our analysis, we first assumed that the stacking order within the domains of adjacent graphene layers is approximately AB- or BA-stacking. As a result, there only exist two types of stacking structures for the unit cells, i.e. ABA-stacking and ABC-stacking. In other words, *R_i_* can have only two values: *R*_ABA_ and *R*_ABC_. Then, based on the idealized sequential atomic registry of the graphene layers in tMLG (Fig. 3b), one can use the SSR model to estimate the effective spreading resistances for the high- and low-conductivity domains of the *m*L/graphite sample, $R_{\rm{H}}^m$ and $R_{\rm{L}}^m$. Thus, the conductivity contrast/ratio between H domain and L domain in *m*L/graphite, *CR^m^*= $R_{\rm{L}}^m/R_{\rm{H}}^m$, can be obtained, and depends only on the sequential atomic stacking states, the relative ratio of *R*_ABA_ and *R*_ABC_ (*β* = *R*_ABA_/*R*_ABC_), and the decaying factor *α*.

By fitting the variations of surface conductivity contrast with the depths of the twisted interface given in Fig. 3c, we found that the theoretical predictions of the SSR model can match well with the experimental data when *α* = 0.6754 and *β* = 0.5633. The magnitude of the decay factor *α* signifies that nearly 90% of the effective spreading resistance is determined by the top five layers of the tMLG. The fitted conductivity ratio of ABA unit to ABC unit (1/*β* = 1.78) is consistent with previous DFT calculation results [35] (more details can be found in Fig. S19), indicating the rationality of our model. Moreover, it is worth noting that our SSR model can capture the abnormally high conductivity contrast on the surface of 2 L/graphite samples. According to our model analysis, for the twisted 2 L/graphite, the two consecutive equivalent resistances in series from the topmost layer downwards in H domain, *R*_1_ and *R*_2_, both are *R*_ABA_, while they are both *R*_ABC_ in L domain. Therefore, the contrast in the equivalent spreading resistance between the H domain and L domain in 2 L/graphite is greater than any other tMLG samples, including the twisted 1 L/graphite sample. Successful prediction of this unique phenomenon also suggests the validity of the SSR model. Since the proposed theoretical model is based on analysis of the internal structures with nearly perfectly stacked interfaces, it is mainly suitable for systems with small twist angles with obvious atomic reconstruction (see further discussion in the section of the details of the SSR model of the supplementary data).

In the above analysis, the stacking state of the adjacent two graphene layers is assumed to be either perfect AB- or BA-stacking; however, this is not exactly the case in real experiments. Moreover, the idealized assumption can only obtain the conductivity values at either H domain or L domain and a continuous conductivity map is lacking. Previous studies have shown that the degree of local interlayer commensurability of layered materials in various domains can be quantified by local registry index (LRI) [40,41], and the interlayer conductivity is positively correlated with the LRI [42]. Therefore, one may obtain the conductivity map of the tMLG using the SSR model if the correlation between the LRI of trilayer graphene and the effective resistance is introduced (see the section of LRI calculation of tMLG in the supplementary data for more details). To do that, we assumed that the resistance of these three layers is proportional to (1/LRI*^γ^*). By fitting the experimental results with the SSR model, considering the local stacking registry, we got *γ* = 1.4581, *α* = 0.8054 (see Figs S20 and S21 in the supplementary data for calculation details). As shown by Fig. 3d and Fig. S22, the theoretically predicted conductivity maps of tMLG devices with different embedded twisted interfaces and varying twist angles agree well with the experimental results shown in Fig. 1b and Fig. S8.

**Figure 4.**
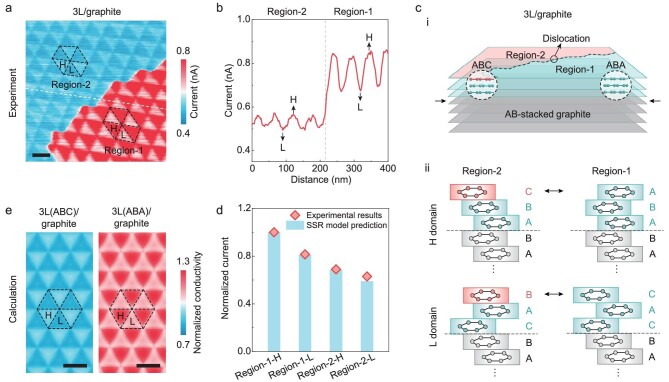
Electrical conductivity of tMLG with crystalline defects. (a) A current map obtained on a twisted 3 L/graphite sample (twist angle of 0.28° ± 0.03°) with a crystalline defect (i.e. a dislocation). Scale bar, 50 nm. (b) A line profile of the current signal obtained along the white dashed line in panel (a). (c) A schematic diagram showing the twisted 3 L/graphite sample with a dislocation within the topmost graphene layer; the dislocation causes a transformation from the initial ABA-stacking to ABC-stacking (panel (i)); the schematics show the idealized stacking structures within Region-1-H, Region-1-L, Region-2-H and Region-2-L (panel (ii)). (d) Conductivities of the four types of domains from experiments, as well as the theoretical predictions from the SSR model with idealized stacking configurations. The conductivities are normalized by the conductivity value of Region-1-H. (e) Conductivity maps calculated using the SSR model with LRI based on the real atomic structures of the twisted ABC-stacked trilayer graphene/graphite (left panel) and the twisted ABA-stacked trilayer graphene/graphite (right panel) systems from MD simulations. Scale bars, 50 nm.

Since the surface conductivity of tMLG is sensitive to the internal interface stacking states, if there exist crystalline defects that can affect the interlayer stacking, these defects may manifest themselves as disturbances to the surface conductivity. Figure 4a shows a surface conductivity map obtained on a twisted 3 L/graphite sample. In contrast to the previous cases showing a relatively uniform two-phase distribution (Fig. 1b), the current map in Fig. 4a is obviously divided into two regions, namely the lower right region with better overall conductivity (Region-1) and the upper left region with lower overall conductivity (Region-2). In both regions, alternately arranged triangular patterns with two different current values can be found, resulting in four distinct conductivity regions, i.e. Region-1-H, Region-1-L, Region-2-H and Region-2-L, as marked in Fig. 4a and b. Despite the existence of two regions with distinct overall conductivities, the triangular moiré patterns and their orientations are roughly continuous across the regions except for a slight disturbance at the boundary. Since this triangular pattern originates from reconstruction of the twisted interface, the continuity of the triangular pattern suggests that the twisted interface is intact and continuous. According to the relative magnitudes of the conductivities of the four regions in 3 L/graphite, it can be determined that there exists a crystalline discontinuity (likely a dislocation) within the topmost surface layer (detailed discussions can be found in Fig. S23). The dislocation will cause a misalignment of the carbon atom positions on both sides of the defect line, converting the original ABA-stacking state into ABC-stacking as schematically depicted in Fig. 4c(ⅰ). Then the resultant stacking structures of Region-1-H, Region-1-L, Region-2-H and Region-2-L can be inferred, as shown in Fig. 4c(ⅱ). This hypothesis was further confirmed by SSR model calculations, whose predictions of the conductivity values (Fig. 4d and Fig. S24) and distribution of the conductivity pattern (Fig. 4e) are in good agreement with the experimental measurements.

## CONCLUSIONS

In conclusion, we found that atomic reconstruction can occur spontaneously for small-angle tMLG structures even when the twisted interface is embedded deep inside. Reconstruction of the embedded twisted interface can induce in-plane deformation to the nearby graphene layers with a decaying magnitude as the adjacent graphene layers always try to stay in registry. Our c-AFM experimental results show that the surface conductivity is sensitive to the sequential stacking state of the internal graphene layers of the tMLG sample up to a depth of ∼10 graphene layers. The correlation between the surface conductivity and the internal stacking structure can be quantitatively described by a theoretical model, which has been proven to be applicable even for tMLG samples with crystalline defects. Our findings provide guidance for tuning the electrical properties of 2D materials by using twisted interfaces, and also enable the visualization and determination of internal stacking structures of tMLG based on simple surface conductivity measurements.

## METHODS

### Sample preparation

The samples were fabricated by the water-assisted transfer method as reported in the literature [35,43]. First, multilayer graphene was freshly cleaved from bulk graphite (purchased from Shanghai Onway Technology Co., Ltd.) and deposited on SiO_2_/Si substrate in ambient conditions by scotch tape. Second, the multilayer graphene was lifted by a small piece of polydimethylsiloxane (PDMS) with the help of a drop of ultra-pure water. Then the multilayer graphene was stacked on a thick graphite flake with a small twist angle ${\theta }_0$ to form the tMLG sample. Typically, the top multilayer graphene was partially laid on the bottom graphite flake with a small twist angle, and partially laid on the SiO_2_/Si substrate to facilitate Raman spectrum measurement (see Fig. S1 for details). The thickness of the mechanically exfoliated top multilayer graphene was identified by both AFM and Raman spectroscopy (Figs S1 and S2). Using this method, we were able to obtain top multilayer graphene flakes with thicknesses of 1∼10 layers and fabricate tMLG samples with controlled twisted interfaces embedded at prescribed depths. We used the period of the moiré pattern *L* to estimate the twist angle ${\theta }_0$ according to the following expression, $L = \sqrt {3a} /(2\sin ({\theta }_0/2))$, where *a* = 1.42 Å is the C−C bond length of graphene.

### Sample measurements

The c-AFM measurements were carried out in an Asylum Research Cypher AFM with conductive probes in ambient conditions (temperature ∼26°C, relative humidity ∼50%). A constant voltage bias was applied between the sample and the probe. The typical spring constant and resonance frequency of the conductive probe coated with Ti/Ir (ASYELEC.01-R2, Asylum Research) are 2.8 N/m and 75 kHz, respectively. To exclude the possibility of an extra moiré pattern in top multilayer graphene created during the transfer process, we also carried out c-AFM measurements on suspended graphene near the graphite edge without the bottom graphite substrate (Fig. S3). In our c-AFM experiments, measurements were intentionally conducted in the inner regions of graphene far away from the graphite edge, where the strain induced by the SiO_2_/Si substrate was minimized (Fig. S4). The Raman spectra were obtained using a Horiba LabRAM HR Evolution Raman microscope with a 532 nm He-Ne laser as the excitation source.

### Molecular dynamics simulations

We built the large rectangular supercells of twisted multilayer graphene/AB-stacked graphite systems, which are periodic in the lateral directions. Here, a 10-layer AB-stacked graphene slab was used to simulate the graphite substrate and a certain number (1∼10) of AB-stacked graphene layers were placed on the graphite substrate with a twist angle of 0.3° to simulate the twisted multilayer structure. An additional twisted trilayer ABC-stacked graphene/AB-stacked graphite model was built to explore the effect of dislocation observed in experiments. The intralayer interaction within the graphene layers was computed via the second-generation reactive empirical bond order (REBO) potential [44]. The interlayer interactions between the graphene layers were described via the registry-dependent interlayer potential (ILP) [45–47] with refined parametrization [48,49], and were implemented in the large-scale atomic/molecular massively parallel simulator (LAMMPS). More details are shown in the section of MD simulation of the atomic reconstruction process of the supplementary data.

## Supplementary Material

nwad175_Supplemental_Files
