# Supplementary material for: Deducing the internal interfaces of twisted multilayer graphene via moiré-regulated surface conductivity
Source: Natl Sci Rev. 2023 Jun 19;10(8):nwad175. doi: 10.1093/nsr/nwad175 (PMC10361741; doi:10.1093/nsr/nwad175)
Supplement: nwad175_Supplemental_Files [file nwad175_supplemental_files.zip › Supplementary data.pdf]

Supplementary material for

Deducing the internal interfaces of twisted multilayer graphene via moiré-regulated surface conductivity

Huan Wang[1,†], Sen Wang[2,†], Shuai Zhang[1,*], Mengzhen Zhu[1], Wengen Ouyang[2,3,*] and Qunyang Li[1,4,*]

[1]Applied Mechanics Laboratory, Department of Engineering Mechanics, Tsinghua University, Beijing 100084, China;

[2]Department of Engineering Mechanics, School of Civil Engineering, Wuhan University, Wuhan 430072, China;

[3]State Key Laboratory of Water Resources & Hydropower Engineering Science, Wuhan University, Wuhan 430072, China;

[4]State Key Laboratory of Tribology in Advanced Equipment, Tsinghua University, Beijing 100084, China

***Corresponding authors.** E-mails: qunyang@tsinghua.edu.cn; shuaizhang@tsinghua.edu.cn; w.g.ouyang@whu.edu.cn

[†]Equally contributed to this work.

This supplementary material contains the following sections:

1. Sample fabrication and experimental characterization

2. MD simulation of the atomic reconstruction process

3. The details of the SSR model

4. Local Registry Index (LRI) calculation of tMLG

5. Effect of crystalline defect on the surface conductivity of 3L/graphite

References

1. Sample fabrication and experimental characterization

The twisted multilayer graphene (tMLG) samples were fabricated by the water-assisted transfer method(1, 2). During the sample preparation, part of the top multilayer graphene was intentionally stacked on the SiO_2/Si substrate in order to facilitate the subsequent Raman and c-AFM measurements, as shown in Fig. S1(a). In c-AFM measurement, a bias voltage was applied between the bottom graphite flake and the conductive tip. The thickness of top multilayer graphene was identified by both AFM height image and Raman spectroscopy. Fig. S1(b) shows the optical images of the typical tMLG samples with varying thicknesses of the top multilayer graphene; Fig. S1(c) shows the Raman spectra of the top multilayer graphene flakes. According to the literature(3), the ratio of the integrated intensities of the G and 2D peak monotonically increases as graphene thickness increasing. Therefore, we used the ratio of the integrated intensities of the G and 2D peak of graphene to initially identify the number of graphene layer as shown in Fig. S1(d). Based on the ratio, the number of graphene layers could be estimated, which was then further confirmed by the topographic measurements using AFM as shown in Fig. S2.

To exclude the possibility of extra moiré pattern in top multilayer graphene created during the transfer process, we conducted c-AFM measurements in two different regions as schematically shown in Fig. S3(a), *i.e.* graphene stacked on graphite substrate (Area-1) and nearby suspended graphene without the bottom graphite substrate (Area-2), to explore the origin of moiré pattern. Fig. S3(b) and (c) show the local current maps of Area-1 and Area-2 measured on a typical sample. Since the moiré patterns can only be observed in Area-1 but not in Area-2, we inferred that the moiré pattern is resulting from the twisted interface between the top multilayer graphene and the bottom graphite substrate.

In addition, the SiO_2/Si substrate adhesion might have a certain impact on deformation of the multilayer graphene. In our work, the surface of the SiO_2/Si substrate is amorphous in nature and is relatively rough (with a root-mean-square roughness of 0.2 nm) compared with the atomically-flat graphite substrate. When graphene is laid on the SiO_2/Si substrate, graphene is known to be adsorbed physically on the surface with weak interaction(4). Therefore, the strain in the multilayer graphene

induced by the SiO_2/Si substrate adhesion is not expected to large(5, 6). Moreover, during the transfer process of the multilayer graphene flakes, the flakes are first stacked on the graphite substrate with a certain twist angle, and then brought into contact with the SiO_2 surface later. Such transfer process can also help minimize the strain of graphene induced by the SiO_2 substrate. However, since the multilayer graphene is partially laid on graphite and partially laid on SiO_2/Si substrate, the sudden drop in height at the edge of graphite may induce extra deformation of the multilayer graphene. To explore this potential impact, we conducted c-AFM measurements at two regions both near the edge and far away from the edge. An example is schematically shown in Fig. S4(a), where Area-1 is 1500 nm away from the graphite edge and Area-2 is closer to the edge. The local current maps obtained on Area-1 and Area-2 are shown in Fig. S4(b) and (c). One can see that the moiré pattern of the region far away from the graphite edge is relatively uniform in size and regular in shape, whereas the moiré pattern of the region around the graphite edge exhibits significant distortion. The transition in the moiré patterns suggests that the substrate adhesion around the graphite edge can indeed cause extra strain to the graphene but its magnitude decays when the location moves far away from the graphite edge. In our c-AFM experiments, measurements were intentionally conducted in the inner regions of graphene far away from the graphite edge, where the strain induced by the SiO_2/Si substrate was minimized.

The typical current maps of tMLGs with top graphene of 1, 2, 3, 4, 6, 7, 8 and 10 layers are shown in Fig. S5, some of which have been shown in the main text. To make a better comparison of the conductivity contrast between the high-conductivity domain and the low-conductivity domain, the color scale bars of the current images in Fig1b and Fig. S5 are fixed at the same level. It can be seen that, when the thickness of the top graphene layer is increased, the bright-dark contrast between H domain and L domain becomes gradually weakened in general. The corresponding twist angles for the individual samples in Fig. S5 range from 0.1° to 0.35°. However, when the color bars are set with the same range, the conductivity contrast between the H domain and the L domain is not so obvious when the twisted interface is embedded more than 6 layers below the surface. So we adjusted the range of the color bars individually for each sample in order to identify the positions of different domains as shown in Fig. S6.

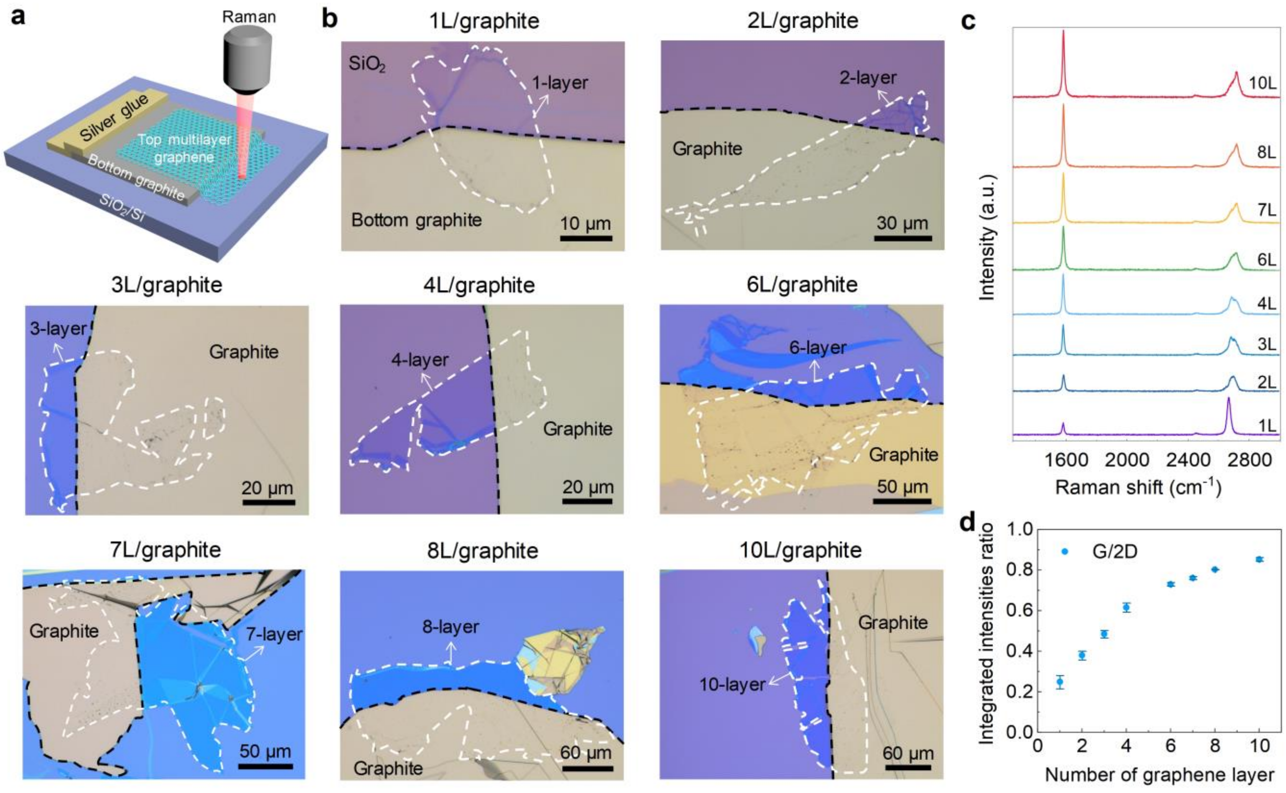

Fig. S1 (a) A schematic diagram of the Raman spectroscopy measurement of the tMLG samples. **(b)** Optical images of the typical tMLG samples, including 1L/graphite, 2L/graphite, 3L/graphite, 4L/graphite, 6L/graphite, 7L/graphite, 8L/graphite, and 10L/graphite. The top multilayer graphene was marked by the white dotted frame, and the border of the bottom graphite substrate was marked with the black dotted curve. **(c)** The Raman spectra of the top multilayer graphene flakes. **(d)** The ratio of the integrated intensities of the G and 2D peaks for samples with different number of graphene layers.

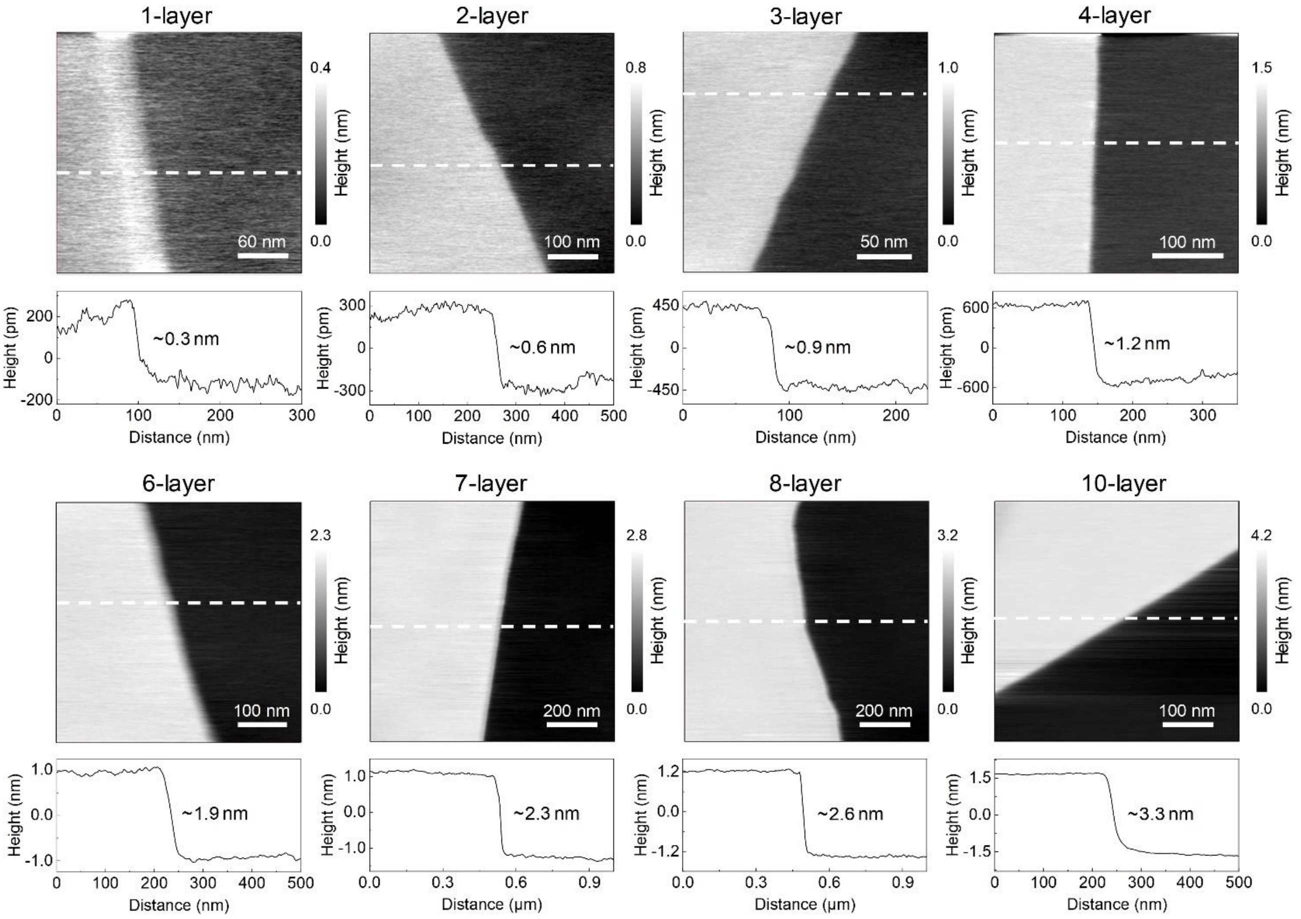

Fig. S2 AFM height data of various tMLG samples, including 1L/graphite, 2L/graphite, 3L/graphite, 4L/graphite, 6L/graphite, 7L/graphite, 8L/graphite, and 10L/graphite. Height line profiles obtained along the white dashed lines of the corresponding top panels.

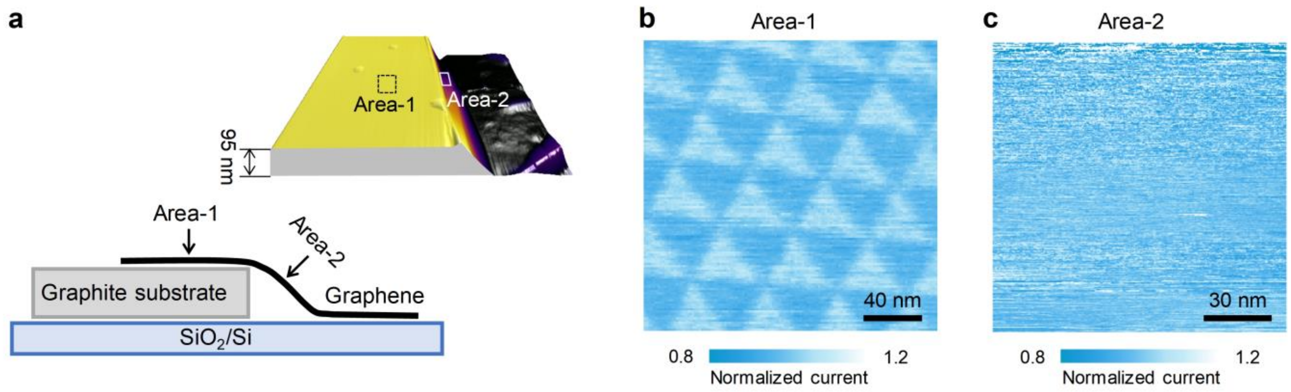

Fig. S3 (a) A schematic diagram showing the c-AFM experiments, the inset in the upper right corner is a 3D height image of a 3L/graphite sample. **(b)**-**(c)** Typical current images obtained in Area-1 and Area-2, whose positions are marked in the inset of (a). The reported current values are normalized by the averaged current value of the corresponding images. The current image obtained in Area-2 shows a very homogeneous surface conductivity, suggesting that the multilayer graphene sheets were not twisted or defective.

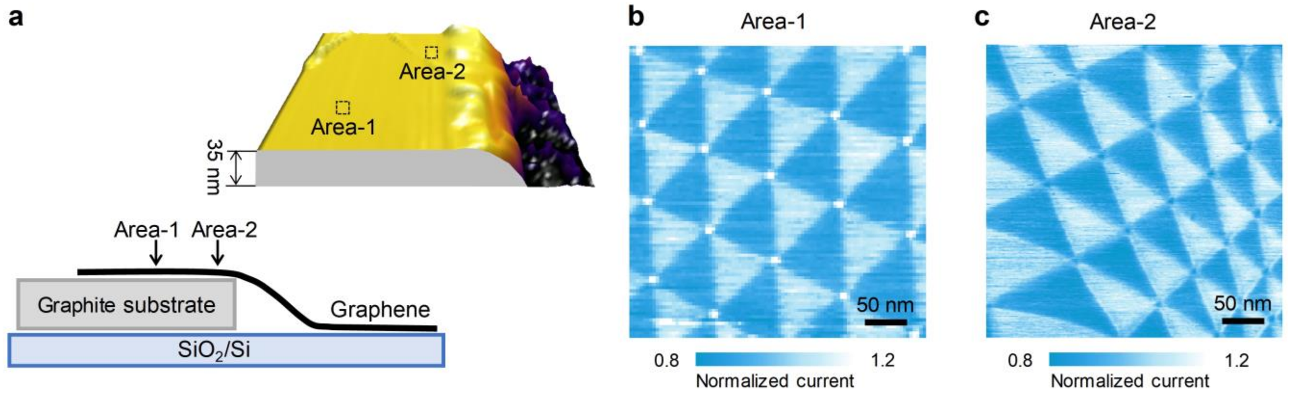

Fig. S4 (a) A schematic diagram showing c-AFM measurements at different locations of graphene, the inset in the upper right corner is a 3D height image of a 1L/graphite sample. **(b)-(c)** Current images obtained on Area-1 and Area-2, whose positions are marked in the inset of (a). The reported current values are normalized by the averaged current value of the corresponding images.

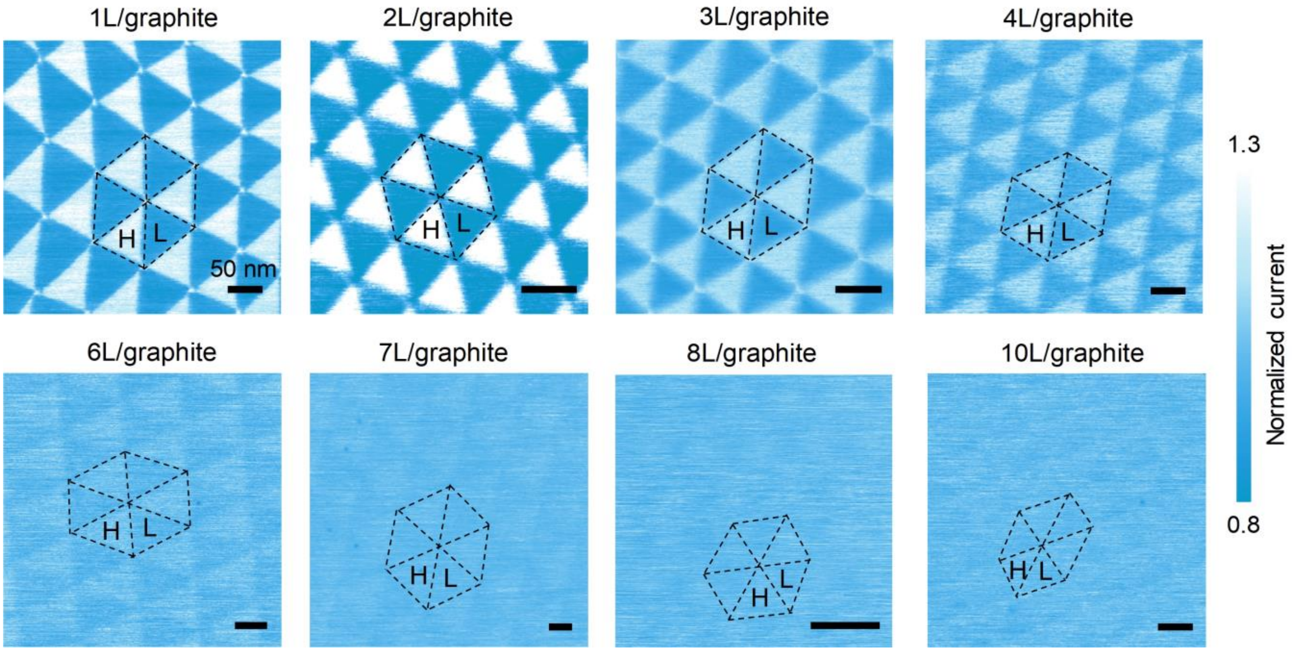

Fig. S5 Typical current maps obtained from the twisted 1L/graphite (with a twist angle of 0.16°± 0.02°), 2L/graphite (0.25°± 0.01°), 3L/graphite (0.20°± 0.02°), 4L/graphite (0.18°± 0.01°), 6L/graphite (0.13°± 0.02°), 7L/graphite (0.11°± 0.01°), 8L/graphite (0.35°± 0.01°) and 10L/graphite (0.21°± 0.04°), respectively. The reported current values are normalized by the averaged current values of the corresponding images. The hexagonal moiré unit cells are highlighted by the black dotted triangles, where L and H represent the low- and high-conductivity domains, respectively. The color bar ranges are the same for all the current images presented in Fig. S5. Scale bars, 50 nm.

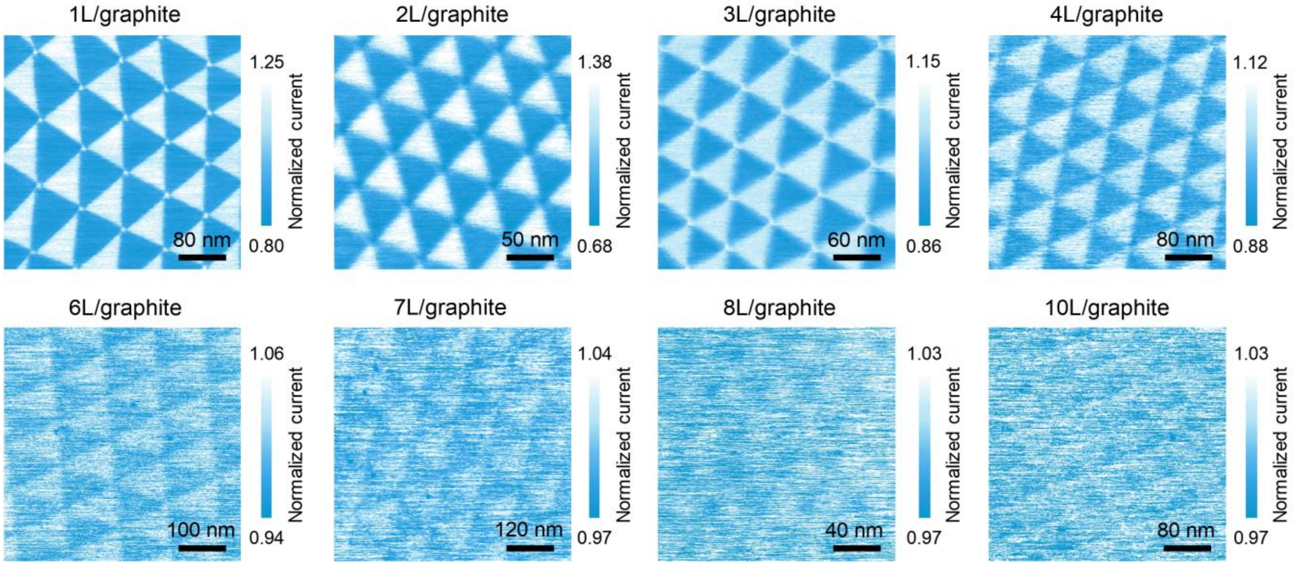

Fig. S6 Raw current figures with independent color bar ranges corresponding to the current images in Fig. S5.

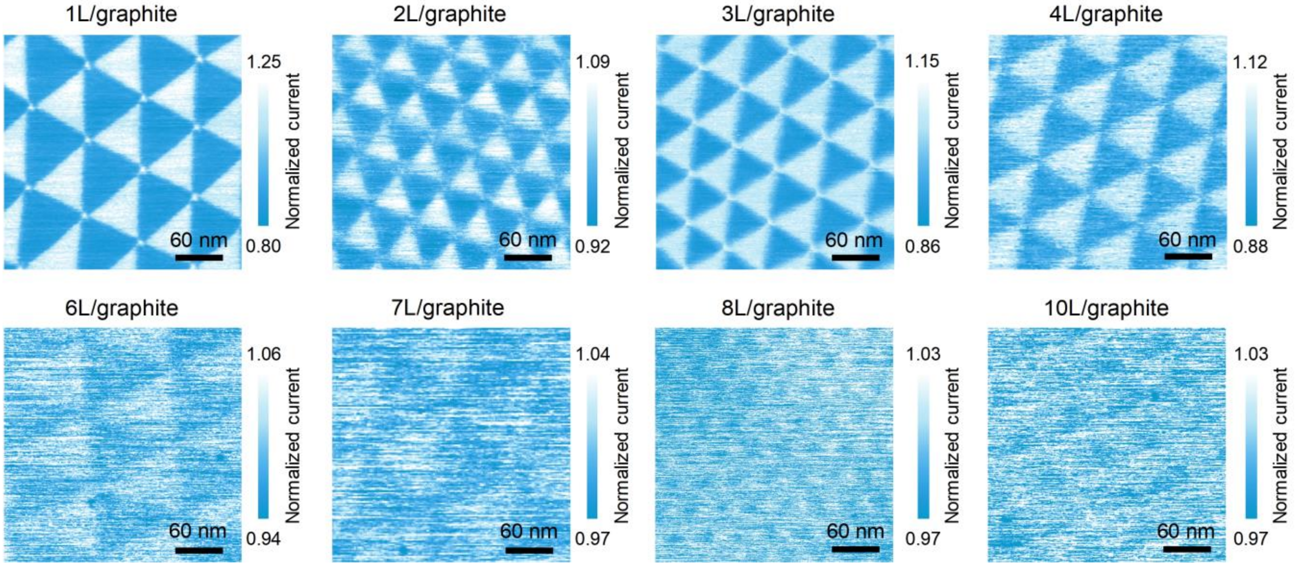

Fig. S7 Current images with same sizes from the twisted 1L/graphite (with a twist angle of 0.16 ° ± 0.02 °), 2L/graphite (0.25 ° ± 0.01 °), 3L/graphite (0.20 ° ± 0.02 °), 4L/graphite (0.18 ° ± 0.01 °), 6L/graphite (0.13 ° ± 0.02 °), 7L/graphite (0.11 ° ± 0.01 °), 8L/graphite (0.35 ° ± 0.01 °) and 10L/graphite (0.21 ° ± 0.04 °), respectively.

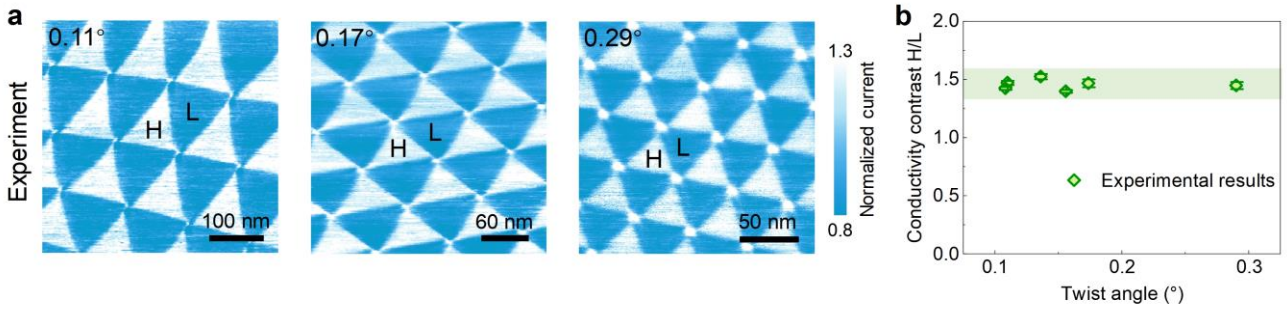

Fig. S8 Effect of twist angle on conductivity contrast of different domains. **(a)** The current images measured on twisted 1L/graphite samples with three twist angles of 0.11 °± 0.01 °, 0.17 °± 0.02 ° and 0.29 °± 0.01 °. The current values are normalized by the averaged current values of the corresponding images. **(b)** The conductivity ratio between H domain and L domain as a function of the twist angle obtained from the experimental measurements. From the 2D current maps, one can see that as the twist angle increases from 0.11 ° to 0.29 °, the conductivity contrast between H domain and L domain barely changes. This indicates that, at small twist angles, the conductivity contrast is not sensitive to the twist angle.

2. MD simulation of the atomic reconstruction process

2.1 Geometry optimization

We built the laterally-periodic rectangular supercells of tMLG systems by following the method outlined in Ref. (7), whereas fixed boundary conditions are applied in the *z*-direction with large enough vacuum size (2 nm). Here, a 10-layer AB-stacked graphene slab was used to simulate the graphite substrate and a certain number (1~10) of AB-stacked graphene layers were placed on the graphite substrate with a twist angle of 0.3 °to simulate the twisted multilayer structure. An additional twisted trilayer ABC-stacked graphene/AB-stacked graphite model was built to explore the effect of dislocation observed in experiments. The lateral dimensions of all systems are 47.09 nm $\times$ 81.56 nm. Doubling this size (94.18 nm $\times$ 81.56 nm) gives almost identical results, which indicates the periodic supercell is large enough to get converged results (Fig. S9). The intra-layer interaction within the graphene layers was computed via the second generation of REBO potential(8). The interlayer interactions between the graphene layers were described via the registry-dependent ILP(9-11) with refined parametrization(12, 13), which were implemented in LAMMPS. The models were initially optimized by using the FIRE algorithm(14) with a threshold force value of 10^{-6} eV ·$Å^{-1}$. To alleviate the residual stress, the whole system was first heated up to 1000 K and then cooled down to 300 K during 100,000 steps using the Nosé-Hoover thermostat with a time-step of 0.25 fs, followed by thermal equilibration at 300 K and zero pressure for another 100,000 steps with a time-step of 1 fs, where the pressure was controlled by the Nosé-Hoover barostat. After the above process, the system was further optimized by using the FIRE algorithm with a threshold force value of 10^{-6} eV ·$Å^{-1}$, followed by a combination of the conjugate gradient (CG) and box/relax algorithm (with a force convergence criterion of 10^{-4} eV ·$Å^{-1}$) for 10 cycles. Finally, the system was optimized again by using the FIRE algorithm with a threshold force value of 10^{-6} eV ·$Å^{-1}$. The above optimization procedure ensures full relaxation of the residual stress in the structures (Fig. S10).

2.2 Convergence tests

2.2.1 The effect of supercell lateral dimensions

To check the size effect of the simulation box, we built a larger 3L/graphite supercell (double the supercell along the *x*-direction, as shown in Fig. S9(a) and (c)) and optimized the structures with the same protocol. The results show that the optimized configurations, primarily represented by the atomic in-plane rotation angle θ_{R} (Fig. S9(a) and (b)) and the out-of-plane displacement (Fig. S9(c) and (d)) maps at the graphene layer adjacent to the twisted interface, are almost identical, confirming that the used supercell is large enough.

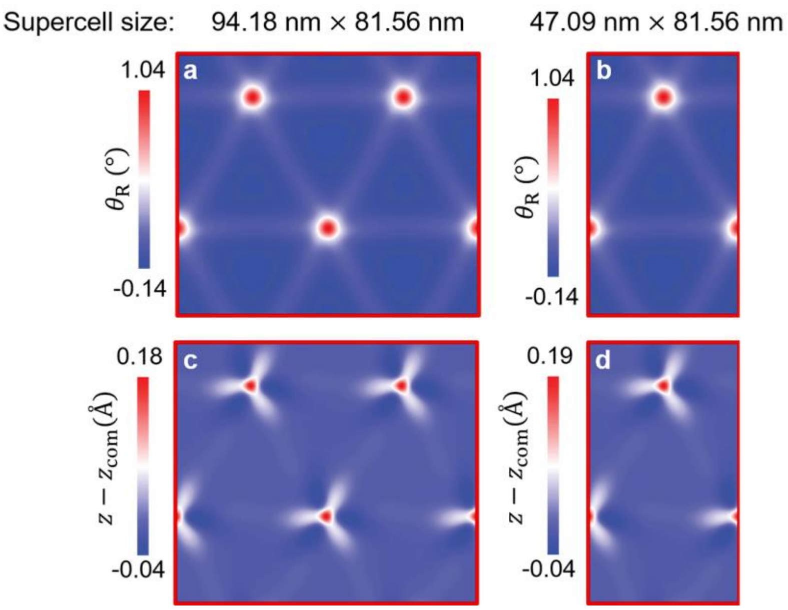

Fig. S9 The effect of supercell lateral dimensions. The atomic rotation angle θ_R **(a)-(b)** and out-of-plane displacement **(c)-(d)** at graphene layer above the twist interface (*i.e.* 1st L) of the 3L/graphite with different supercell size. θ_R is obtained by calculating the relative rotation angle before and after relaxation. θ_R is positive when it rotates in the direction of the twist angle and negative otherwise. The out-of-plane displacement in panel (c) and (d) is calculated as the vertical height with respect to the center of mass of the corresponding layers. The simulation box is marked by the red rectangle in each panel.

2.2.2 Convergence tests of the repetition times of the box relaxation

As mentioned in Sec. 2.1, when we optimized the models, the simulation box of supercell was relaxed with ten cycles by using the box/relax algorithm after NVE and NPT ensemble simulations to eliminate the possible residual stresses. As shown in Fig. S10, the energy, supercell dimensions and in-plane pressure converge after several relaxation cycles to within $3\times10^{-6}\,\mathrm{eV}$, $2\times10^{-8}\,\mathrm{nm}$, and $3\times10^{-8}\,\mathrm{GPa}$ respectively, which indicates that the model is well optimized and the external stress induced by the lateral periodic boundary is effectively eliminated.

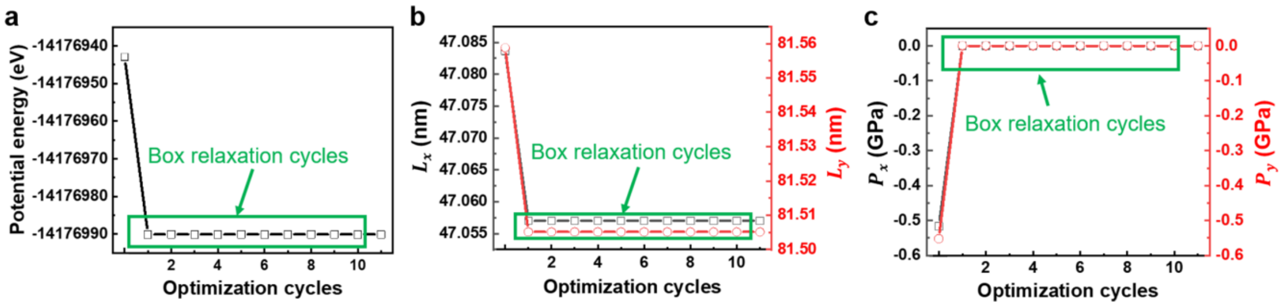

Fig. S10 Convergence test of optimization cycles for the 3L/graphite. Change of the **(a)** potential energy, **(b)** simulation box dimensions and **(c)** in-plane stress on the box during the optimization. The left and right axes in (b) and (c) represent the size of the simulation box and the in-plane stress of the system along *x* (open black squares) and *y* (open red circles) directions, respectively.

2.3 Atomic structures of the reconstructed *m*L/graphite

The atomic in-plane rotation angle and out-of-plane displacement maps of *m*L/graphite ($m = 1, 2, 3, 6, 10$) are shown in Fig. S12-Fig. S16, respectively. We can find that the most significant in-plane and out-of-plane deformation occur at the twisted interface (1st L and B1 L) for all *m*L/graphite models. Meanwhile, the in-plane rotation and out-of-plane displacement at the twisted interface can propagate to the surrounding graphene layers (although the amplitude gradually decays, as shown in Fig. S17). The multilayer graphene above the twisted interface also imposes a constraint on the atomic reconstruction, which can be observed in Fig. S17. Namely, the in-plane rotation $\theta_{\mathrm{R}}^{\mathrm{domain}}$ and the magnitude of out-of-plane fluctuation ($z_{\mathrm{max}} - z_{\mathrm{min}}$) of the 1st L in the fewer-layer top graphene systems ($m = 1, 2, 3$) are larger than those in thicker top graphene systems ($m = 6, 10$).

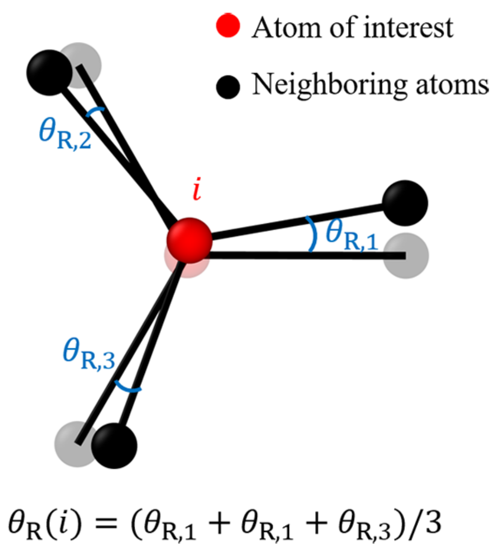

Fig. S11 The schematic diagram showing the definition of the atomic in-plane rotation angle θ_R. Three C-C bonds exist between the calculated atom i (red) and the neighboring atoms (black) are rotated after the atomic reconstruction occurs, and the averaged value of the rotation angles of the three bonds was defined as θ_R of the calculated atom i. The transparent and opaque circles represent the atoms before and after structural reconstruction, respectively.

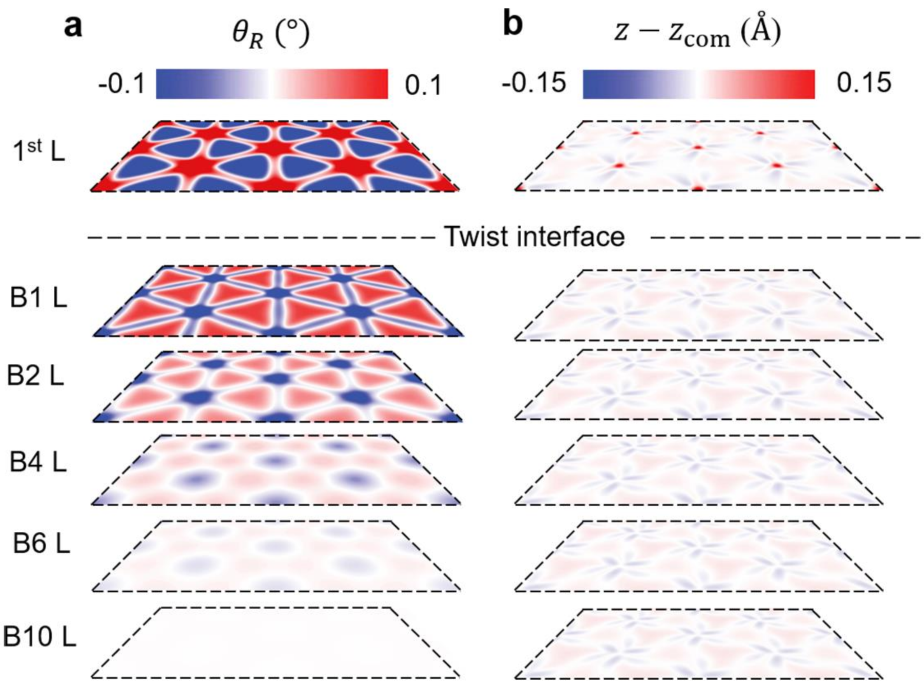

Fig. S12 Distributions of the atomic in-plane rotation angle θ_R **(a)** and out-of-plane displacement **(b)** for 1L/graphite. θ_R is obtained by calculating the relative rotation angle before and after relaxation. θ_R is positive when it rotates in the direction of the twist angle and negative otherwise. The out-of-plane displacement ($z - z_{com}$) in panel (b) is calculated as the vertical height with respect to the center of mass of the corresponding layers.

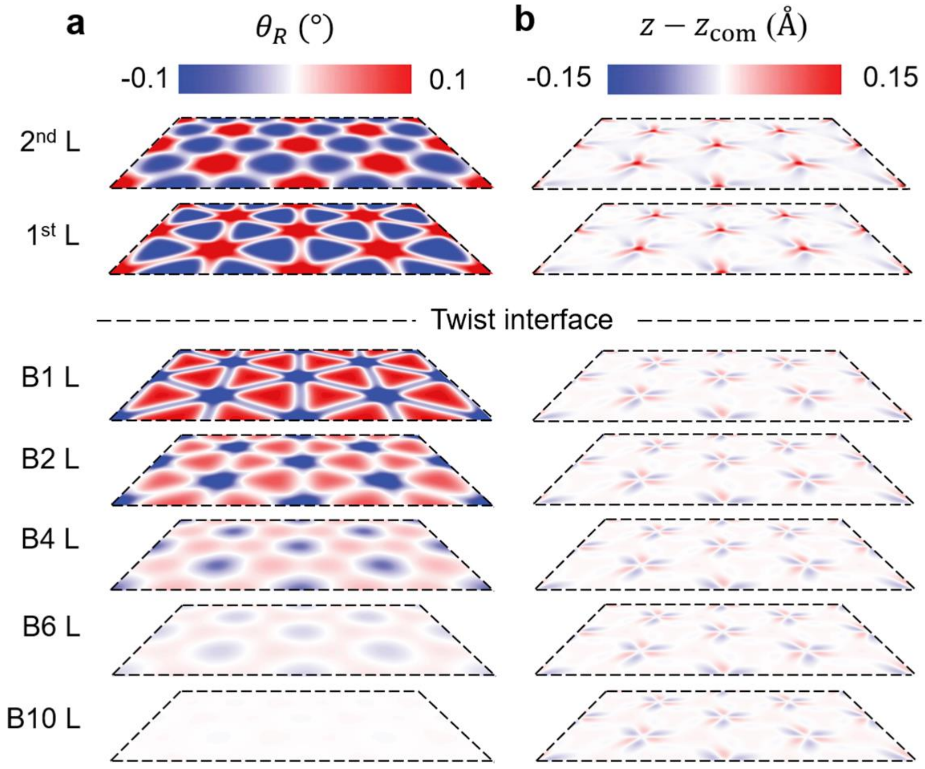

Fig. S13 Distributions of the atomic in-plane rotation angle θ_R **(a)** and out-of-plane displacement **(b)** for 2L/graphite. The definitions of θ_R and out-of-plane displacement are the same as Fig. S12.

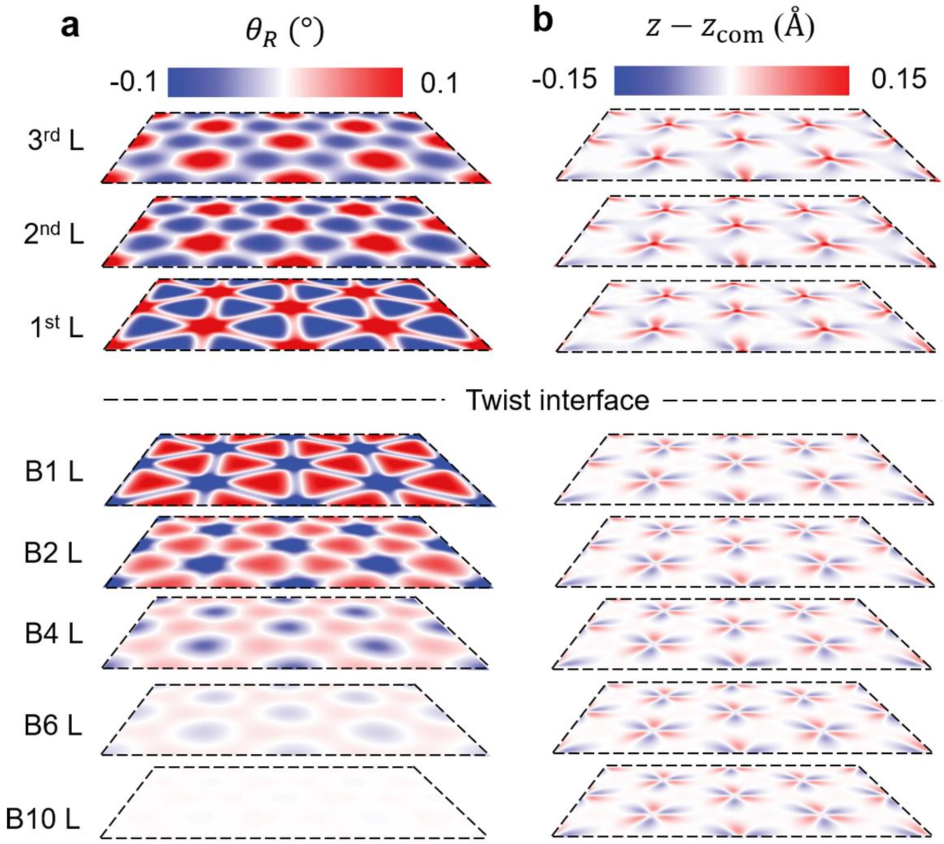

Fig. S14 Distributions of the atomic in-plane rotation angle θ_R **(a)** and out-of-plane displacement **(b)** for 3L/graphite. The definitions of θ_R and out-of-plane displacement are the same as Fig. S12.

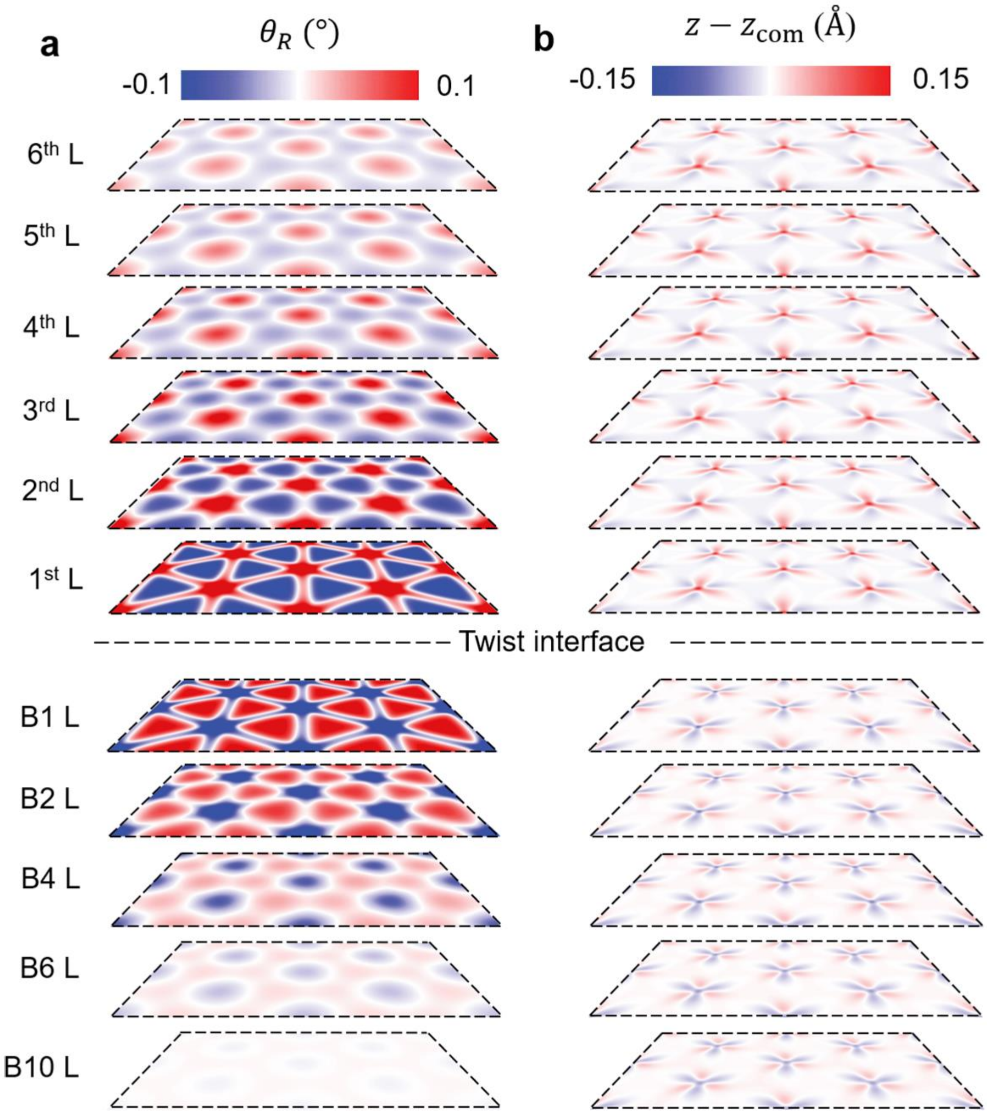

Fig. S15 Distributions of the atomic in-plane rotation angle θ_{R} **(a)** and out-of-plane displacement **(b)** for 6L/graphite. The definitions of θ_{R} and out-of-plane displacement are the same as Fig. S12.

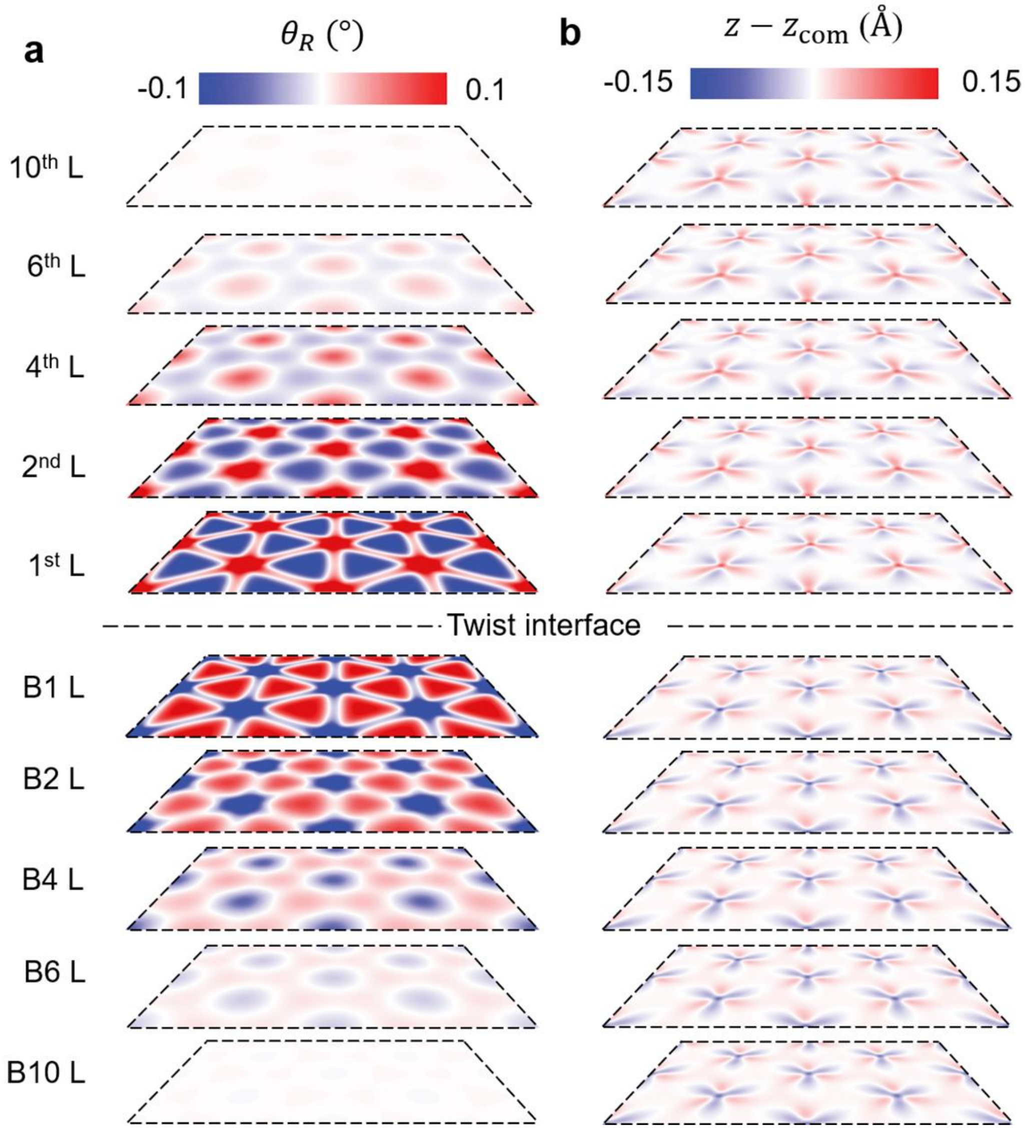

Fig. S16 Distributions of the atomic in-plane rotation angle θ_R **(a)** and out-of-plane displacement **(b)** for 10L/graphite. The definitions of θ_R and out-of-plane displacement are the same as Fig. S12.

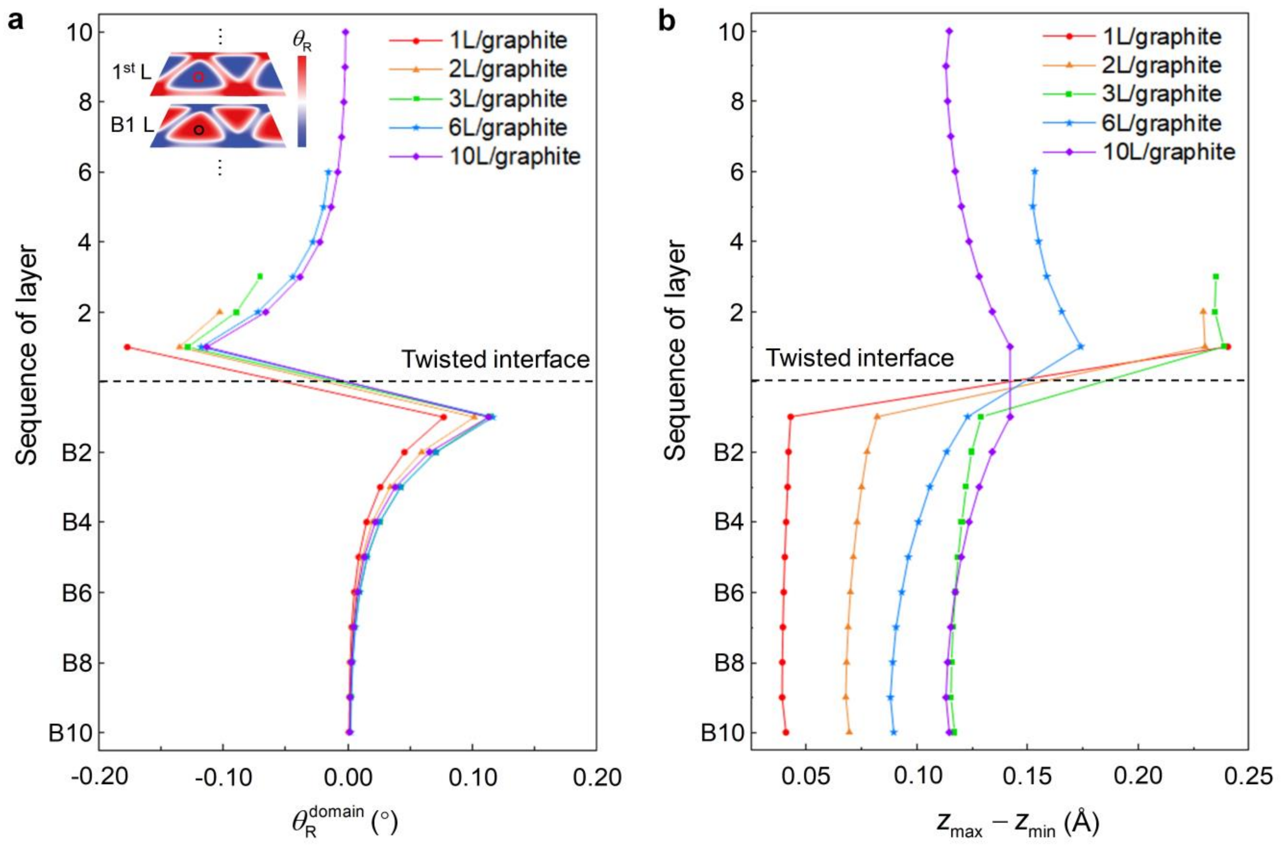

Fig. S17 The averaged values of θ_R^{domain} in the center of the domains **(a)** and the peak-to-valley values ($z_{max} - z_{min}$) of the out-of-plane displacement **(b)** of the individual graphene layer for *m*L/graphite systems. θ_R^{domain} are the averaged values of θ_R for all atoms within the center area of the AB/BA domains (within a radius of 2 nm), as marked by the circles in the inset of panel (a). ($z_{max} - z_{min}$) is the magnitude of the out-of-plane displacement ($z - z_{com}$), which represents the difference between the maximum and minimum values of ($z - z_{com}$) for individual graphene layer.

3. The details of the SSR model

3.1 The derivation of the formula for the SSR model

In our experiments, the current flows from the region on the surface where the tip touches the sample, through the internal graphene layers one by one while gradually spreading out to the bottom. In view of this vertical electrical conduction, the graphene layers are connected in series. Moreover, the vertical conductivity through a given graphene layer is not only related to itself, but also affected by the adjacent layers and the overall stacking structure. Previous DFT calculations and experimental results have shown that the vertical conductivity through a graphene layer is essentially determined by a trilayer unit and the ABA- and ABC-stacked trilayers are the two basic units with distinct electrical behaviors(15, 16). Therefore, in our series spreading resistance model, for a tMLG structure with N graphene layers, R_i (i=1,2,3…N-2) represents the equivalent resistance of individual unit cell containing three neighboring graphene layers and these unit cells are nested in series as shown in Fig. S18. Since each unit cell contains two interfaces, when R_i are nested in series, the internal interfaces have different degrees of repeated calculation. For convenience, the interfaces are numbered from the top to the bottom with the top one being called Interface-1 and the bottom one being called Interface-(N-1). When summing up R_i, Interface-1 is calculated only once from R_1; Interface 2 is calculated twice from R_1 and R_2; Interface 3 is calculated twice from R_2 and R_3; similarly, Interfaces-4 to Interface-(N-2) are also calculated twice; the interface (N-1) is calculated once from R_{N-2}. If we multiply R_i (i=2,3…N-3) by 0.5 and multiply R_i (i=1 and N-2) by 1.0, then Interface-3 to Interface-(N-3) will be counted once, and Interface-1 and Interface-(N-1) are still counted once, while Interface-2 and Interface-(N-2) will be counted 1.5 times. To further account for the 0.5 times repetitive computation on Interface-2 and Interface-(N-2), we multiply R_i (i=1 and N-2) by 0.75 instead of 1.0. Such operation is based on the assumption that Interface-2 and Interface-(N-2) contribute half of the vertical conductance of R_1 and R_{N-2}. Therefore, the coefficient of R_i is 0.75 for the surface units and 0.5 for the inner units when considering the case of repeated calculation.

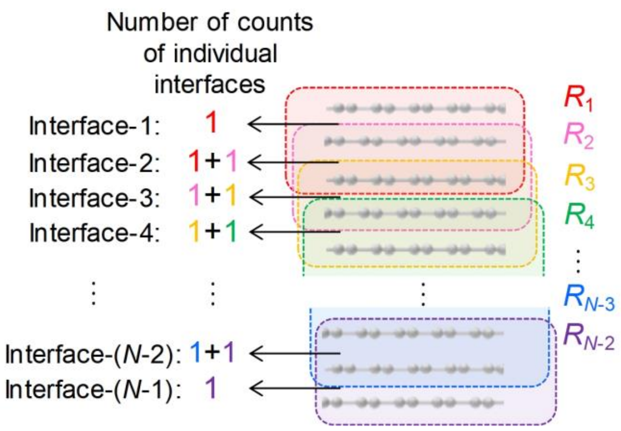

Fig. S18 A schematic diagram showing the process for counting the conductance of different interfaces. The numbers on the left show the number of counting for individual interfaces when the conductance are summed directly without taking account the repeated calculations.

3.2 The electrical transport calculation of ABA- and ABC-stacked trilayer graphene

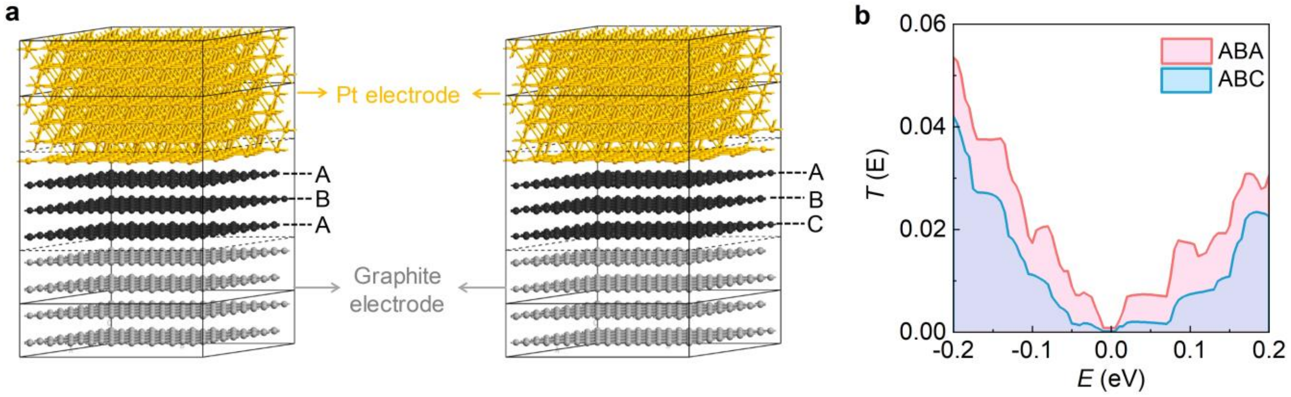

Fig. S19 The electrical transport calculation of ABA- and ABC-stacked trilayer graphene reproduced from reference(1). **(a)** A schematic diagram showing the DFT calculation model for ABA (left panel) and ABC (right panel) stacking. **(b)** Calculated transmission spectra of ABA and ABC stacking under zero external pressure. Since the electrical conductivity is correlation with the integral of the transmission spectra, by calculating the transmission spectra area ratio in the range of -0.2 eV to 0.2 eV, we estimated the conductivity ratio of ABA to ABC to be 1.68.

3.3 Discussions of the SSR model

For slightly larger twist angles, the proposed SSR model in our work would be still applicable as long as there is obvious atomic reconstruction. However, when the twist angle is larger than the characteristic angle θ_c which has been predicted to be around 1°, the atomic registry within the domains starts deviating appreciably from perfect AB-stacking and the transition in registry from one domain to the neighboring domain also becomes rather gradual(17-19). At the same time, the domain shape will change from triangle to hexagon gradually and it will be difficult to clearly/ambiguously define the H and L domains. For such samples with large twist angles, the two graphene layers at the twisted interface gradually decouple, and the equilibrium structures of the graphene layers do not differ substantially from the simple rigid-body rotation as the atomic reconstruction no longer occurs. Because the proposed theoretical model is based on analysis of the internal structures with nearly perfectly-stacked interfaces, it is not suitable for the systems with large twist angles.

According to the MD simulations, the individual layers in tMLG sample are indeed undulated. However, the magnitude of the out-of-plane fluctuation ($z_{max} - z_{min}$) are typically very small (about 0.02 Å); even the maximum value, which occurs at the domain vertices, does not exceed 0.25 Å. This undulation height is much smaller compared with the initial graphene interlayer spacing (about 3.35 Å). Based on the literature(20), the electrical conductivity across a graphene interface is much more sensitive to the atomic registry of the layers (*i.e.* the local stacking) rather than the weak out-of-plane fluctuation. Since our model mainly focuses on the electrical conductivity inside the relatively flat domains, we neglected the influence of the height fluctuation in our model analysis.

4. Local Registry Index (LRI) calculation of tMLG

4.1 Trilayer graphene Case

The rationality of the trilayer graphene as the basic unit for calculating conductivity is discussed in the main text. Moreover, the electrical conductivity of graphene layers is known to be directly related to its stacking structure(20), which can be quantified by the LRI(21, 22). Hence, the first step is to calculate the LRI of the trilayer graphene structure, or more specifically, the LRI of the top layer of a trilayer graphene structure. Here, we take the top three layers of 1L/graphite (*i.e.*, 1st L, B1 L and B2 L shown in Fig. S12) as an example. The LRI value of each atom in 1st L is defined as the sum of the LRI values of 1st L and the two layers below (B1 L and B2 L), which can be expressed by the formula (S1):

$$\mathrm{LRI}_{1^{st}\,\mathrm{L}} = \mathrm{LRI}_{1^{st}\,\mathrm{L/B1L}} + \mathrm{LRI}_{1^{st}\,\mathrm{L/B2L}}, \qquad \text{(S1)}$$

where $\mathrm{LRI}_{1^{st}\,\mathrm{L/B1L}}$ and $\mathrm{LRI}_{1^{st}\,\mathrm{L/B2L}}$ represent the LRI values of 1st L and B1 L, 1st L and B2 L, respectively. The $\mathrm{LRI}_{1^{st}\,\mathrm{L/B1L}}$ and $\mathrm{LRI}_{1^{st}\,\mathrm{L/B2L}}$ are normalized to range from 0 (designating no overlap with underlying atoms) to 1 (indicating a fully eclipsed atomic center)(20). Noting that in our calculation, the LRI of the optimized/relaxed configuration of 1L/graphite is calculated.

The LRI map is obtained by coloring each atom in 1st L according to the value of its LRI, which shows two distinct triangular domains with high (domain 1) and low (domain 2) LRI values, respectively (see Fig. S20(a)). Further analysis of the enlarged side view of the LRI map shows that the LRI values in domain 1 show atomic fluctuation (see Fig. S20(b)) in the range of [0.922,1.998]. This phenomenon results from the ABA-like stacking structure of domain 1, which leads to some atoms on 1st L have a large overlap area with atoms in both B1 L and B2 L while the others can only have a large overlap area in B2 L. The stacking structure of domain 2 is ABC-like stacking and the fluctuation of LRI values are relatively small (see Fig. S20(c)), which is in the range of [0.863, 1.211]. This is because each atom on 1st L in this domain can only have a large overlap area with the atom in one of the lower two layers.

According to the above method, we can calculate the LRI of the other three adjacent layers (for example, B1 L, B2 L and B3 L, etc.) of 1L/ graphite and the similar algorithm can be extended to other *m*L/graphite systems.

4.2 SSR model calculation with LRI

Previous study has shown that the local interlayer conductance is positively correlated with LRI[2]. Hence, we assume that the resistance of three adjacent graphene layers is proportional to ($1/\mathrm{LRI}^{\gamma}$). Noting that the experimental current value measured at each position is actually an average effect of a certain contact region. We define LRI_{mn} as a smear-out continuous function of the position, *i.e.*, *x*- and *y*-coordinates. For each determined position (x_m, y_n), the average value of the LRI of all atoms within a radius of 2 nm around (x_m, y_n) is taken as the value of LRI_{mn} (see Fig. S20(d)). Fig. S20(e) shows the map of LRI_{mn} (also taking the top layer of 1L/graphite as an example) with a mesh size of 2 Å. The LRI_{mn} map is similar to that of atomic LRI map (Fig. S20(a)), while its value is in the range of 0.96 and 1.49. In the same way, we can obtain the LRI_{mn} map of any three adjacent layers of *m*L/graphite.

We assume that the equivalent resistance of ith three adjacent graphene layers from the topmost layer can be expressed as

$$R_i = R_0 / \left(\mathrm{LRI}_{mn,i}\right)^{\gamma}, \tag{S2}$$

where $R_i\,(i = 1, 2, 3...)$ represent the equivalent resistance; $\mathrm{LRI}_{mn,i}$ represent the LRI_{mn} for the ith three adjacent layers from the topmost layer, and R_0 represent the resistance of the ABC-stacked tri-layer. We can get the expression for the resistance of whole graphene layers (R) at any position (x_m, y_n) of *m*L/graphite by using the series spreading resistance (SSR) model considering local stacking registry, namely, combining Eq. 1 and Eq. S2. The parameters α and γ can be fitted according to the experimental results (conductivity ratio of the high and low conductivity domains) shown in Fig. 1c using the least-square-fit method (Fig. S21).

Since the actual LRI is not uniformly distributed, the choice of the regions may influence the results. In our calculation, the center regions in domains 1 and 2 are selected by offsetting the edges

of the triangle domains with a distance of d towards the center (Fig. S20(e)). The effect of the choice of d on the fitting results are discussed in Sec. 4.3.

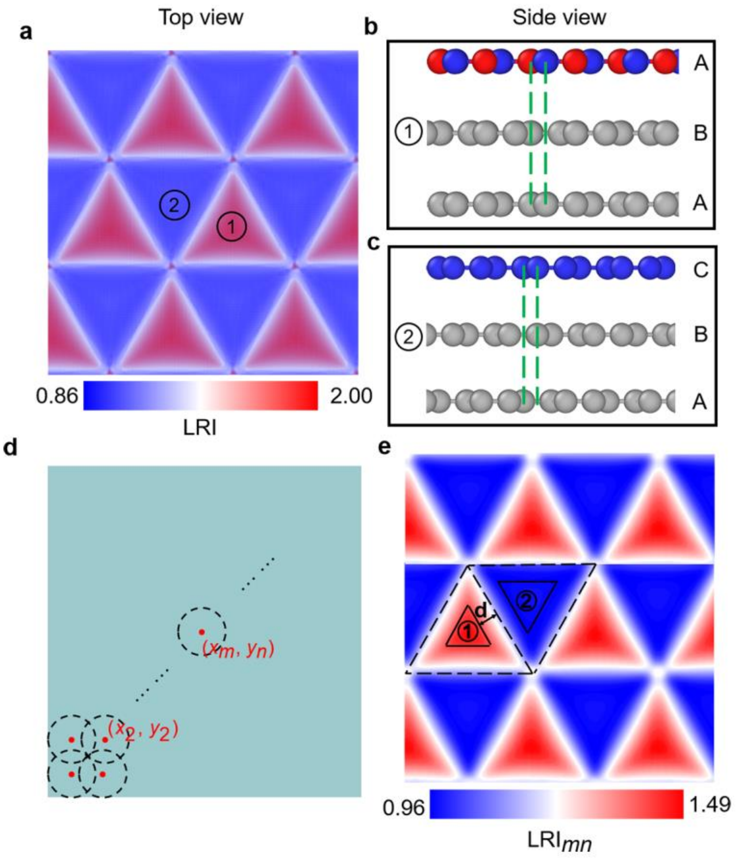

Fig. S20 (a) The LRI map of 1st L of 1L/graphite. Panels **(b)** and **(c)** show the enlarged side view of region 1 and 2, respectively. The atoms of 1st L are colored by the LRI value and the atoms of B1 L and B2 L are shown in (b) and (c) to represent of the corresponding stacking state. **(d)** A schematic diagram for calculating the smear-out value of the LRI in a local region (LRI_{mn}). The atoms in the black circles with a radius of 2 nm are used to calculate the value of LRI_{mn} and the red dots represent the center of the corresponding circles. **(e)** The LRI_{mn} map of 1st L of 1L/graphite. Region 1 and 2 are high and low conductivity domains respectively. d is the offset distance of high conductivity domain (dotted triangle) relative to the corresponding half of Moiré lattice (solid triangle).

4.3 Parameter fitting with experimental results

The effect of the offset distance d (Fig. S20(e)) on the fitting values of α and γ is considered in the fitting process. The results show that the choice of d has a minor effect on the fitting parameter values (Table S1). The conductivity contrasts between H domain and L domain in *m*L/graphite calculated by the SSR model considering LRI are in good agreement with the experimental data even when different values of d are used (Fig. S21).

The interlayer conductivity at any position (x_m, y_n) of *m*L/graphite can be calculated by the SSR model after the values of α and γ are determined ($\alpha = 0.8054$, $\gamma = 1.4581$). Based on it, we can calculate the conductivity maps of *m*L/graphite, as shown in Fig. 3(d) and Fig. 4(e) in the main text.

Table S1 The effect of the offset distance d (Fig. S20(e)) on the fitting results of α and γ.

d (nm)	12	10	8	6
α	0.7744	0.7803	0.7898	0.8054
γ	1.3825	1.4024	1.4341	1.4581

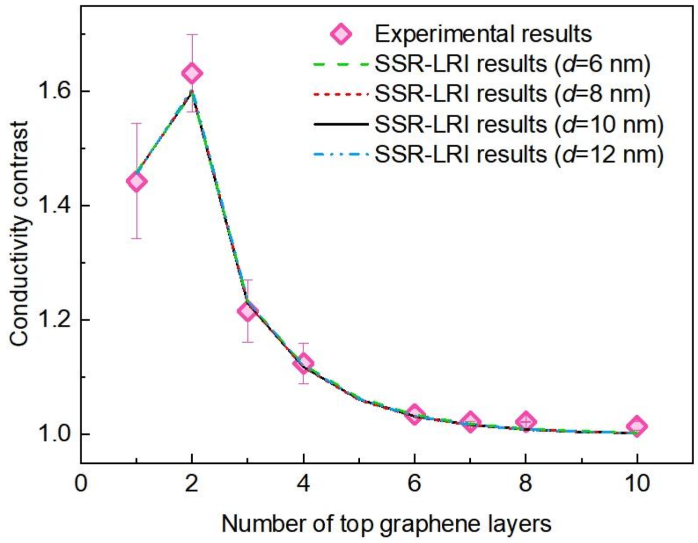

Fig. S21 Comparison between the experimental results and the SSR model predictions based on LRI with different values of d .

4.4. SSR-LRI predicted conductivity

We have carried out SSR-LRI calculations to explore the effect of twist angle on conductivity contrast with the fitting values of $\alpha = 0.8054$ and $\gamma = 1.4581$, $d = 6$ nm. As shown in Fig. S22(a) and (b), one can see that as the twist angle increases from 0.16° to 0.5°, the conductivity contrast between H domain and L domain barely changes, consistent with the experimental results as shown in Fig. S8. This is reasonable because that the local conductivity is closely related to the local atomic registry, while for very small twist angle, spontaneous atomic reconstruction would result in nearly commensurate stacking within the H and L domain. Therefore, the conductivity contrast is almost independent of the twist angle for small twist angles. However, once the twist angle is larger than the characteristic angle θ_c which has been predicted to be around 1°, the atomic registry within the domains starts deviating appreciably from perfect AB-stacking, the conductivity contrast would also be different from the case with small twist angles(17-19). Therefore, the conductivity contrast is expected to be insensitive to the twist angle when the twist angle is small, which is the case in our experiments since most the twist angles of our samples are lower than 0.35°.

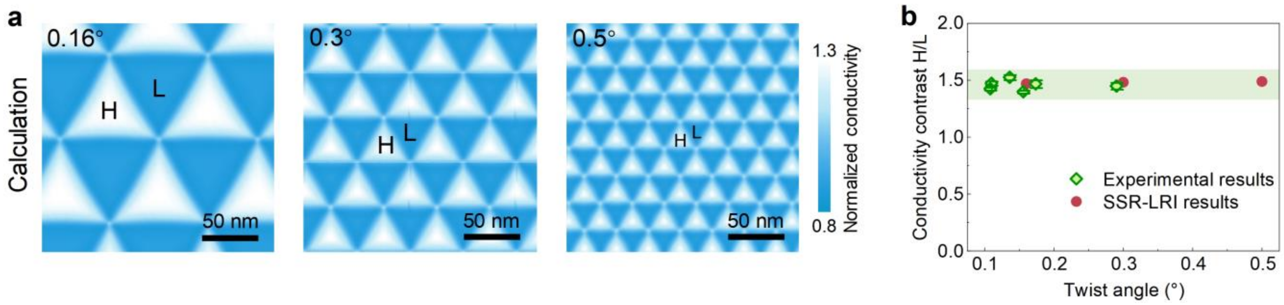

Fig. S22 Effect of twist angle on conductivity contrast of different domains. **(a)** Calculated conductivity maps using the SSR model with LRI based on the real atomic structures of the twisted 1L/graphite systems with twist angles of 0.16 °, 0.3 ° and 0.5 ° from MD simulations. The calculated conductivity values are normalized by the averaged values of the corresponding images. **(b)** The conductivity ratio between H domain and L domain as a function of the twist angle obtained from experimental measurements (green) and the SSR-LRI calculations (red).

5. Effect of crystalline defect on the surface conductivity of 3L/graphite

If the crystalline defect (*e.g.* dislocation) exists in the twisted interface layer, the corresponding positions of different domains in the one side of the defect boundary will be different from that in the other side; in this case, the moiré superlattice in Region-1 and Region-2 will no longer be continuous across the defect boundary, which clearly contradicts the experimental results. Therefore, the following will mainly discuss the situation where the crystalline defect/dislocation is not within the twisted interface layer. Firstly, if the dislocation exists only in the second topmost layer of the top 3-layer graphene slab, then the defect will transform the pristine ABA-stacking into ACA-stacking (Fig. S23(a) ⅰ) and the ideal stacking structures of Region-1-H, Region-1-L, Region-2-H and Region-2-L can be inferred (Fig. S23(a) ⅱ). The normalized conductivities of the four regions from the SSR model are compared with the experimental results in Fig. S23(a)ⅲ. It can be seen that the theoretically predicted conductivity of Region-2-L is greater than that of Region-2-H, and the conductivities of Region-2-H and Region-2-L are both greater than that of Region-1-L based on the ideal stacking structures, which do not agree with the conductivity contrast obtained experimentally. Secondly, if the dislocations exist both in the second topmost layer and the topmost layer, then the defect will transform the pristine ABA-stacking into ACB-stacking (Fig. S23(b) ⅰ); in this case, the ideal stacking structures(Fig. S23(b) ⅱ) and the theoretically predicted conductivities (Fig. S23(b)ⅲ) of the four regions can be obtained. However, the theoretically predicted conductivity of Region-2-L is greater than that of Region-2-H (Fig. S23(b)ⅲ) in contradiction to the experimental observation. Thirdly, if the dislocation exists in the B2 layer (Fig. S23(c) ⅰ), the theoretically predicted conductivity of Region-1-L will be the smallest (Fig. S23(c)ⅲ), which is also inconsistent with the experimental results. Actually, any dislocation in B*n* layer ($n \neq 1$) will cause the conductivity of Region-1-L to be smaller than that of Region-2-H, in contradiction to the experimental observation. Therefore, combining the analyses above and the discussion about Fig.4 in the main text, it can be concluded that the dislocation can only exist within the topmost graphene layer.

In addition, we used the SSR model based on LRI to calculate the conductivity ratio of 3L/graphite when the crystalline defect exists in the top graphene layer; and the results are also in good agreement with the experimental observations (Fig. S24).

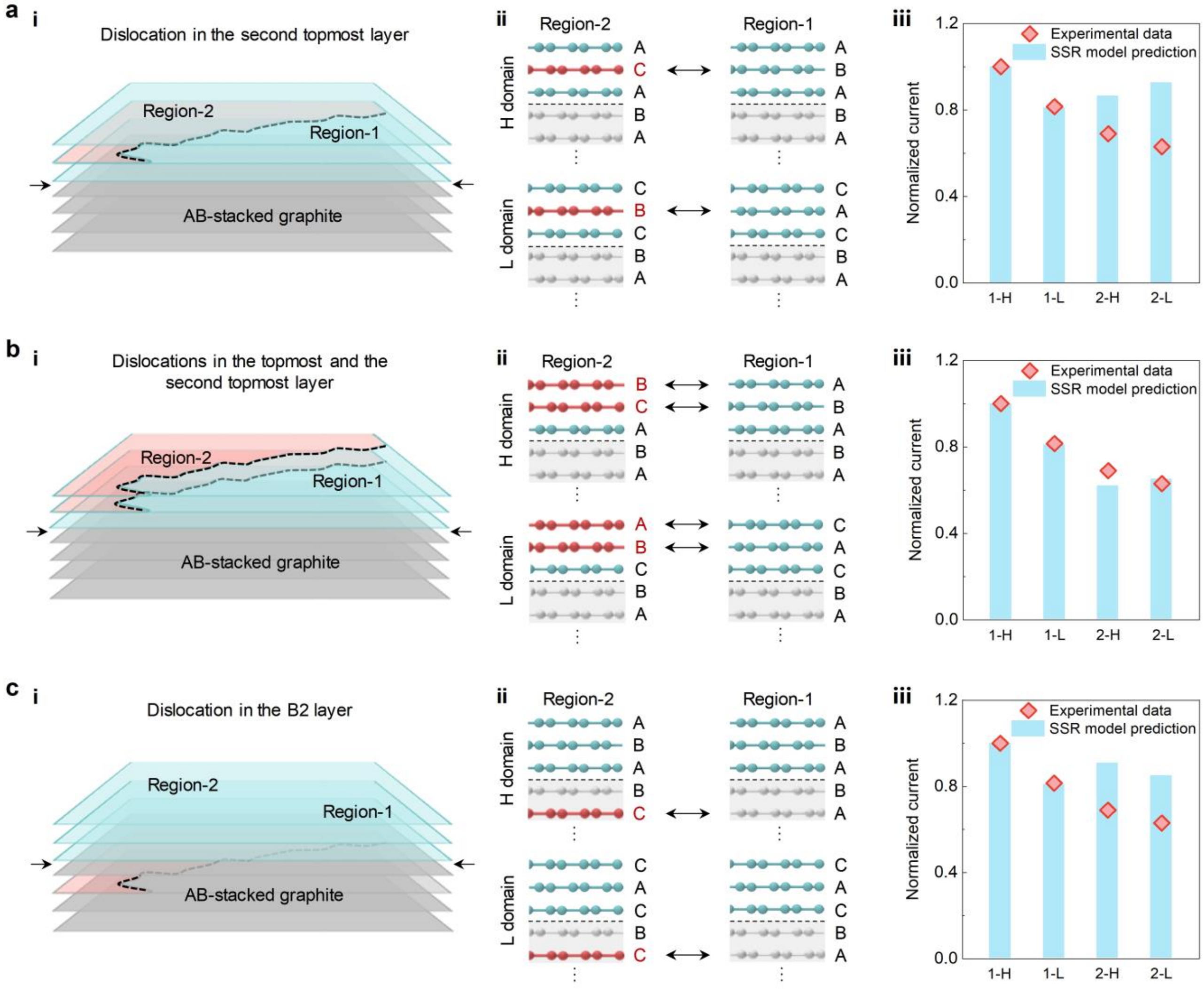

Fig. S23 The stacking structures and the predicted conductivities by the SSR model of Region-1-H, Region-1-L, Region-2-H and Region-2-L in 3L/graphite with crystalline defects/dislocations existing in the second topmost layer **(a)**; in the second topmost layer and the topmost layer **(b)**; and in the B2 layer **(c)**. The panels (i), (ii) and (iii) in (a)-(c) have the same meaning. Schematic diagrams showing the twisted 3L/graphite sample with crystalline defects in different layers (panel i); the schematics show the ideal stacking structures within Region-1-H, Region-1-L, Region-2-H and Region-2-L (panel ii); the comparison of the normalized conductivities of the four regions calculated from the SSR model and the experiment results in Fig. 4 of the main manuscript (panel iii).

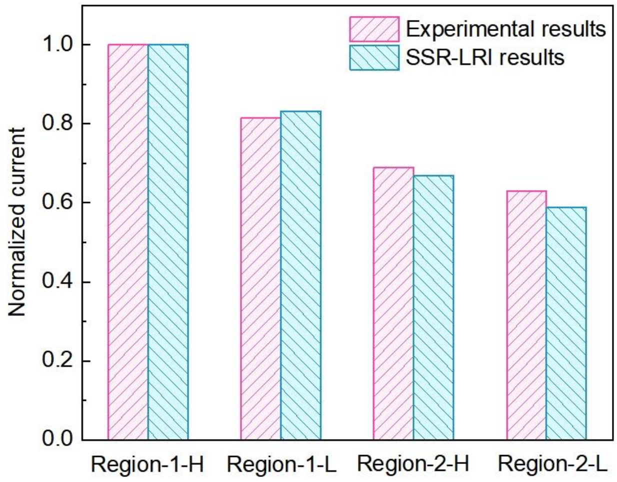

Fig. S24 The normalized conductivities of the four areas from the SSR model based on LRI and the experiments for 3L/graphite system with a crystalline defect (*i.e.* a dislocation) in Fig.4a of the main manuscript.

References

1. Zhang, S, Xu, Q, Hou, Y, *et al.* Domino-like stacking order switching in twisted monolayer–multilayer graphene. *Nature Materials*. 2022; **21**(6): 621-6.

2. Hou, Y, Ren, X, Fan, J, *et al.* Preparation of Twisted Bilayer Graphene via the Wetting Transfer Method. *ACS Applied Materials & Interfaces*. 2020; **12**(36): 40958-67.

3. Graf, D, Molitor, F, Ensslin, K, *et al.* Spatially Resolved Raman Spectroscopy of Single- and Few-Layer Graphene. *Nano Letters*. 2007; **7**(2): 238-42.

4. Fan, XF, Zheng, WT, Chihaia, V, *et al.* Interaction between graphene and the surface of SiO2. *Journal of Physics: Condensed Matter*. 2012; **24**(30): 305004.

5. Teague, ML, Lai, AP, Velasco, J, *et al.* Evidence for Strain-Induced Local Conductance Modulations in Single-Layer Graphene on SiO2. *Nano Letters*. 2009; **9**(7): 2542-6.

6. Nemes-Incze, P, Kukucska, G, Koltai, J, *et al.* Preparing local strain patterns in graphene by atomic force microscope based indentation. *Scientific Reports*. 2017; **7**(1): 3035.

7. Ouyang, W, Qin, H, Urbakh, M, *et al.* Controllable Thermal Conductivity in Twisted Homogeneous Interfaces of Graphene and Hexagonal Boron Nitride. *Nano Letters*. 2020; **20**(10): 7513-8.

8. Brenner, DW, Shenderova, OA, Harrison, JA, *et al.* A second-generation reactive empirical bond order (REBO) potential energy expression for hydrocarbons. *Journal of Physics: Condensed Matter*. 2002; **14**(4): 783-802.

9. Leven, I, Azuri, I, Kronik, L, *et al.* Inter-layer potential for hexagonal boron nitride. *The Journal of Chemical Physics*. 2014; **140**(10): 104106.

10. Leven, I, Maaravi, T, Azuri, I, *et al.* Interlayer Potential for Graphene/h-BN Heterostructures. *Journal of Chemical Theory and Computation*. 2016; **12**(6): 2896-905.

11. Maaravi, T, Leven, I, Azuri, I, *et al.* Interlayer Potential for Homogeneous Graphene and Hexagonal Boron Nitride Systems: Reparametrization for Many-Body Dispersion Effects. *The Journal of Physical Chemistry C*. 2017; **121**(41): 22826-35.

12. Ouyang, W, Mandelli, D, Urbakh, M, *et al.* Nanoserpents: Graphene Nanoribbon Motion on Two-Dimensional Hexagonal Materials. *Nano Letters*. 2018; **18**(9): 6009-16.

13. Ouyang, W, Azuri, I, Mandelli, D, *et al.* Mechanical and Tribological Properties of Layered Materials under High Pressure: Assessing the Importance of Many-Body Dispersion Effects. *Journal of Chemical Theory and Computation*. 2020; **16**(1): 666-76.

14. Bitzek, E, Koskinen, P, Gähler, F, *et al.* Structural Relaxation Made Simple. *Physical Review Letters*. 2006; **97**(17): 170201.

15. Yankowitz, M, Wang, JIJ, Birdwell, AG, *et al.* Electric field control of soliton motion and stacking in trilayer graphene. *Nature Materials*. 2014; **13**(8): 786-9.

16. Bao, W, Jing, L, Velasco, J, *et al.* Stacking-dependent band gap and quantum transport in trilayer graphene. *Nature Physics*. 2011; **7**(12): 948-52.

17. Gargiulo, F, Yazyev, OV. Structural and electronic transformation in low-angle twisted bilayer graphene. *2D Materials*. 2018; **5**(1): 015019.

18. Zhang, K, Tadmor, EB. Structural and electron diffraction scaling of twisted graphene bilayers. *Journal of the Mechanics and Physics of Solids*. 2018; **112**: 225-38.

19. Yoo, H, Engelke, R, Carr, S, *et al.* Atomic and electronic reconstruction at the van der Waals interface in twisted bilayer graphene. *Nature Materials*. 2019; **18**(5): 448-53.

20. Koren, E, Leven, I, Lörtscher, E, *et al.* Coherent commensurate electronic states at the interface between misoriented graphene layers. *Nature Nanotechnology*. 2016; **11**(9): 752-7.

21. Hod, O. The Registry Index: A Quantitative Measure of Materials′ Interfacial Commensurability. *ChemPhysChem*. 2013; **14**(11): 2376-91.

22. Leven, I, Guerra, R, Vanossi, A, *et al.* Multiwalled nanotube faceting unravelled. *Nature Nanotechnology*. 2016; **11**(12): 1082-6.
